# Curcumin prevents As^3+^-induced carcinogenesis through regulation of GSK3β/Nrf2

**DOI:** 10.1186/s13020-021-00527-x

**Published:** 2021-11-10

**Authors:** Yuan-Ye Dang, Hua Luo, Yong-Mei Li, Yang Zhou, Xiu Luo, Shui-Mu Lin, Shou-Ping Liu, Simon Ming-Yuen Lee, Chu-Wen Li, Xiao-Yan Dai

**Affiliations:** 1 The Fifth Affiliated Hospital, Key Laboratory of Molecular Target & Clinical Pharmacology and the State & NMPA Key Laboratory of Respiratory Disease, School of Pharmaceutical Sciences, Guangzhou Medical University, Guangzhou, 511436 PR China; 2 State Key Laboratory of Quality Research in Chinese Medicine, Institute of Chinese Medical Sciences, University of Macau, Macao, China

**Keywords:** Arsenic, Curcumin, Nrf2, ROS, GSK-3β/β-TrCP, Autophagy, Carcinogenesis

## Abstract

**Background:**

Arsenic (As^3+^) is a carcinogen with considerable environmental and occupational relevancy. Its mechanism of action and methods of prevention remain to be investigated. Previous studies have demonstrated that ROS is responsible for As^3+^-induced cell transformation, which is considered as the first stage of As^3+^ carcinogenesis. The NF-E2 p45-related factor-2 (Nrf2) signaling pathway regulates the cellular antioxidant response, and activation of Nrf2 has recently been shown to limit oxidative damage following exposure to As^3+^

**Methods and results:**

In this study, molecular docking was used to virtually screen natural antioxidant chemical databases and identify molecules that interact with the ligand-binding site of Keap1 (PDB code 4L7B). The cell-based assays and molecular docking findings revealed that curcumin has the best inhibitory activity against Keap1-4L7B. Co-immunoprecipitation (Co-IP) results indicated that curcumin is a potent Keap1 Kelch domain-dependent Nrf2 activator that stabilizes Nrf2 by hindering its ubiquitination. The increased activation of Nrf2 and its target antioxidant genes by curcumin could significantly decrease As^3+^-generated ROS. Moreover, curcumin induced autophagy in As^3+^-treated BEAS-2B via inducing autophagy by the formation of a p62/LC-3 complex and increasing autophagic flux by promoting transcription factor EB (TFEB) and lysosome-associated membrane protein 1 (LAMP1) expression. Knockdown of Nrf2 abolished curcumin-induced autophagy and downregulated ROS. Further studies showed that inhibition of autophagosome and lysosome fusion with bafilomycin a1 (BafA1) could block curcumin and prevented As^3+^-induced cell transformation. These results demonstrated that curcumin prevents As^3+^-induced cell transformation by inducing autophagy via the activation of the Nrf2 signaling pathway in BEAS-2B cells. However, overexpression of Keap-1 showed a constitutively high level of Nrf2 in As^3+^-transformed BEAS-2B cells (AsT) is Keap1-independent regulation. Overexpression of Nrf2 in AsT demonstrated that curcumin increased ROS levels and induced cell apoptosis via the downregulation of Nrf2. Further studies showed that curcumin decreased the Nrf2 level in AsT by activating GSK-3β to inhibit the activation of PI3K/AKT. Co-IP assay results showed that curcumin promoted the interaction of Nrf2 with the GSK-3β/β-TrCP axis and ubiquitin. Moreover, the inhibition of GSK-3β reversed Nrf2 expression in curcumin-treated AsT, indicating that the decrease in Nrf2 is due to activation of the GSK-3β/β-TrCP ubiquitination pathway. Furthermore, in vitro and in vivo results showed that curcumin induced cell apoptosis, and had anti-angiogenesis and anti-tumorigenesis effects as a result of activating the GSK-3β/β-TrCP ubiquitination pathway and subsequent decrease in Nrf2.

**Conclusions:**

Taken together, in the first stage, curcumin activated Nrf2, decreased ROS, and induced autophagy in normal cells to prevent As^3+^-induced cell transformation. In the second stage, curcumin promoted ROS and apoptosis and inhibited angiogenesis via inhibition of constitutive expression of Nrf2 in AsT to prevent tumorigenesis. Our results suggest that antioxidant natural compounds such as curcumin can be evaluated as potential candidates for complementary therapies in the treatment of As^3+^-induced carcinogenesis.

## Introduction

It has been well established that environmental and occupational exposure to arsenic (As^3+^) causes cancers of various organs, including the lung [1–5]. Although the mechanism of As^3+^-induced carcinogenesis remains unknown, As^3+^-induced generation of reactive oxygen species (ROS) is considered to be important. The transcription factor Nrf2 serves as a “master regulator” in response to oxidative/electrophilic stresses and chemical insults through the induction of numerous cytoprotective genes. Therefore, the activation of Nrf2 is considered an important approach to preventing diseases triggered by stress and toxins, including lung carcinogenesis.

Under normal conditions, the levels of Nrf2 protein are maintained at a low level by the E3 ubiquitin ligase Kelch ECH-associating protein 1 (Keap1), which ubiquitinates Nrf2 in the cytoplasm and targets it for degradation by the 26 S proteasome. Two residues, Cys273 and Cys288, appear to be essential for Keap1 to control Nrf2 under both basal and stress conditions, whereas Cys151 is primarily required under conditions of stress [6]. As demonstrated previously, when cells are exposed to exogenous stimulants, such as sulforaphane (SF) and tert-butylhydroquinone (tBHQ), the activity of the Keap1-E3 ubiquitin ligase complex is impaired due to modifications of critical cysteine residues in Keap1, particularly Cys151, leading to stabilization of Nrf2 [7]. Various natural chemicals act as antioxidants and are used for chemoprevention. Some of these natural compounds exhibit their antioxidant effects through activating the Nrf2 pathway. Previous studies have used the luciferase assay under the control of antioxidant response element promoters to show that trans-chalcone, sulforaphane, curcumin, flavone, kahweol, and carnosol have better antioxidant efficacy than tert-butylhydroquinone [8]. Recent studies have shown that antioxidant natural compounds, such as tanshinone I, Withaferin A, and Piperlongumine, are potent Keap1-C151-dependent Nrf2 activators that stabilize Nrf2 by hindering its ubiquitination [9–11].

In contrast, Nrf2 activation in cancer cells also contributes to the promotion of tumor growth in many forms of cancer [12]. Indeed, constitutive activation of Nrf2 has been identified in several types of human cancer cell lines and tumors [13]. Somatic mutations in Keap1 and Nrf2 have been identified in lung, gall bladder, and head and neck tumors. These mutations lead to constitutive activation of Nrf2, followed by the induction of Nrf2 target genes, protective enzymes, and antioxidant proteins, resulting in resistance of tumor cells to oxidative stress, apoptosis, and anticancer agents. Recent studies have identified GSK-3β as a novel regulator of Nrf2 [6, 14]. GSK-3β phosphorylates a group of Ser residues in the Neh6 domain of Nrf2 that overlaps with an SCF/β-TrCP destruction motif (DSGIS, residues 334 to 338) to promote Keap1-independent degradation. Moreover, GSK-3β acts upstream of Fyn kinase, which phosphorylates tyrosine 568 of Nrf2, leading to the nuclear export of Nrf2. Nuclear accumulation of Nrf2 might be induced by the increased inhibitory phosphorylation of GSK-3β (Ser9 and Thr390). Among Nrf2 inducers, flavonoids have been demonstrated to be regulators of the GSK-3β-associated signaling pathway in Nrf2 activation. Puerarin, one of the most extensively studied flavonoids, induces nuclear translocation of Nrf2 and stimulates the expression of Nrf2-dependent genes in APP/PS1 transgenic mice and neurons through activation of the phosphatidylinositol 3-kinase (PI3K)/GSK-3β pathway. Quercetin, the aglycone of hyperoside, improves hippocampus-dependent learning and memory in mice through the PI3K/protein kinase B (AKT)/Nrf2 pathway. Moreover, flavonoid-mediated Nrf2 nuclear translocation might be mediated via the phosphorylation of mitogen-activated protein kinase (MAPK), AKT, and GSK-3β, either in isolation or combination [15].

Previous studies have found that Nrf2 is antioncogenic in early stages of As^3+^-induced cell transformation via the upregulation of antioxidants, which reduces reduce As^3+^-induced ROS. However, once a cell is transformed, Nrf2 is oncogenic by inducing apoptosis resistance. Recent studies have shown that Nrf2 intensifies host defense systems to prevent lung carcinogenesis, but accelerates malignant cell growth after tumor initiation [12]. Therefore, Nrf2 can play dual roles in carcinogenesis. Activation of inducible Nrf2 decreases carcinogenesis, especially in its early stages (cell transformation). Enhancement of Nrf2 activity, which lessens oxidative or mutagenic stress, appears to be beneficial during pre-malignant states. However, constitutively activated Nrf2 can be oncogenic by protecting cancer cells against oxidative stress and chemotherapeutic agents. To develop potential prevention agents, we screened various natural compounds in an attempt to select those with two properties: first, those that can activate Nrf2 and decrease ROS in non-transformed cells to prevent As^3+^-induced cell transformation, and second, those that can inhibit constitutive expression of Nrf2 and enhance ROS and apoptosis in As^3+^-transformed cells to prevent tumorigenesis. As a result, we found a series of antioxidant natural compounds with such dual properties. Among them, curcumin showed the most potential regarding the prevention of As^3+^-induced carcinogenesis.

Curcumin, an acidic polyphenol compound extracted from the roots of plants such as ginger, has good anti-inflammatory and anti-tumor effects. This natural chemopreventive agent, derived from rhizomes of curcuma species, provides antioxidant, anti-tumor, and anti-proliferative efficacy. Curcumin blocks cancer development by modulating multiple signaling pathways. Researchers have previously provided novel perceptions about the mechanisms of curcumin action in gastric cancer cell growth inhibition and its therapeutic strategies for gastric cancer control. Therefore, curcumin could be considered a novel therapeutic strategy to control gastric cancer cell growth [16]. Some studies have emphasized the importance of curcumin in lung cancer treatment and the potential utility of curcumin as a method for improving therapeutic outcome [17–20]. Curcumin suppressed gemcitabine-resistant non-small cell lung cancer cell proliferation and induced apoptosis. Curcumin upregulated the expression of lncRNA-MEG3 and PTEN. MEG3 overexpression has been shown to increase PTEN expression, while MEG3 knockdown decreased PTEN expression in gemcitabine-resistant non-small cell lung cancer cells. Previous studies have also showed that curcumin has potential clinical application in gemcitabine-resistant non-small cell lung cancer treatment for its anti-tumor activity [21]. Furthermore, curcumin also showed anti-inflammatory activity in the kidney. Another study identified curcumin as a common inhibitor of NLRP3 inflammasome activation and revealed that curcumin repressed inflammation [22]. In view of the various advantages and characteristics of curcumin, its anti-tumor effect has attracted the attention of researchers, and has good application prospect in the prevention and treatment of lung cancer.

## Materials and methods

### Chemicals and reagents

Sodium arsenite (Na_2_AsO_2_, CAS:7784-46-5), apocynin, 5,5-dimethyl-1-pyrroline-1-oxide (DMPO), and Annexin V/propidium iodide (PI) were purchased from Sigma (St Louis, MO). Both 5-(and -6)-chloromethyl-2,7-dichlorodihydrofluorescein diacetate, acetyl ester (DCFDA) and dihydroethidium (DHE) were purchased from Molecular Probes (Eugene, OR). Curcumin, luteolin, genistein, and butein were purchased from Sigma-Aldrich (St. Louis, MO). Shikonin, wogonin, plumbagin, bis(2-hydroxybenzal) acetone, phenethyl isothiocyanate, rutin, and piperlongumine were purchased from Cayman Chemical (Ann Arbor, MI). Plasmid DNA encoding human catalase and SOD2 and catalase shRNA were purchased from Origene (Rockville, MD). Antibody against SOD2 was purchased from Millipore (Billerica, MA). Antibodies against catalase (#14097), AKT(#9272), pAKT^Ser473^(#9271), PI3K(#4257), p-PI3K(#4228), C-PARP(#5625), C-Caspase-3(#9664), Bcl-2(#3498), and wortmannin were purchased from Cell Signaling Tech (Danvers, MA).

### Cell culture and treatment

The human lung bronchial epithelial cell line BEAS-2B was obtained from the American Type Culture Collection (Manassas, VA). As^3+^-transformed BEAS-2B cells were generated as described previously [23, 24]. The transformation ability and tumorigenicity of the transformed cells were confirmed by soft agar assay and xenograft assay, respectively [23, 24].

### Plasmids and transfection

The method of pCDNA3.1 FLAG NRF2 (F-Nrf2), HA-tagged Keap1 (H-Keap1), and HA-ubiquitin (H-Ub) overexpression in BEAS-2B cells has been described previously [23, 24]. The mCherry-EGFP-LC3B plasmid and GFP-LC3 plasmid were purchased from Addgene (Cambridge, MA) (18). Control siRNA and siRNAs for knockdown of Nrf2 and GSK3β were obtained from Santa Cruz Biotechnology, Inc. (Santa Cruz, CA). Transfections were performed using Lipofectamine™ 3000 (Invitrogen) according to the manufacturer’s protocol.

### Cell viability assay

Cell viability was determined using 3-(4,5-dimethylthiazol-2yl-2,5-diphenyl tetrazoliumbromide) (MTT) assay. Active mitochondrial dehydrogenases in living cells metabolize MTT to a purple formazan dye, which is measured photometrically at 570 nm using a spectrophotometer, as described previously [25].

### Intracellular ROS determination

ROS generation was examined using the fluorescent dye DCFDA and DHE, respectively, as described previously [23, 24]. The cells were cultured in 6-well plates with 5 × 10^4^ cells/well. The cells were treated with curcumin and/or As^3+^ for 24 h and then incubated with DCFDA or DHE (final concentration, 10 μM) for 30 min at 37 °C. The fluorescence signal was imaged by an Olympus BX53 fluorescence microscope (Center Valley, PA) and the fluorescence intensity was measured using flow cytometry (FACS Calibur, BD Biosciences). The fluorescence intensity of DCF was measured at an excitation wavelength of 492 nm and an emission wavelength of 517 nm. The fluorescence intensity of DHE was measured at an excitation wavelength of 535 nm and an emission wavelength of 610 nm.

### As^3+^-induced cell transformation

BEAS-2B cells were treated with 0.5 μM As^3+^. The medium was replaced with fresh medium every 3 days. After 48 weeks, 1 × 10^4^ cells were suspended in 2 mL culture medium containing 0.35% agar and seeded into 6-well plates with a 0.5% agar base layer, and maintained in an incubator for 4 weeks. The cells were stained with 1 mg/mL iodonitrotetrazolium violet, and colonies greater than 0.1 mm in diameter were scored by microscope examination. As^3+^-transformed cells from anchorage-independent colonies were picked up and continued to grow in DMEM. Passage-matched cells without As^3+^ treatment were used as controls.

### Western blotting

Cell lysates were prepared in ice-cold RIPA buffer (Sigma-Aldrich) with freshly added protease inhibitor cocktail. The lysate was then centrifuged at 12,000 g for 10 min at 4 °C and the supernatant (total cell lysate) was collected, aliquoted, and stored at − 80 °C. Nuclear and cytoplasmic extracts were prepared using an extraction kit from Thermo Scientific (Rockford, IL) according to the manufacturer’s protocol. The protein concentration was determined using Coomassie Protein Assay Reagent (Thermo, Rockford, IL). Approximately 40 μg of cellular proteins were separated through 6–12% SDS polyacrylamide gel, and then transferred to a nitrocellulose membrane (Bio-Rad, Hercules, CA). Nonspecific binding was blocked with 5% fat-free milk in 1 × Tris-buffered saline (TBS) and the membrane was incubated with antibodies, as indicated. Protein bands, detected with horseradish peroxidase-conjugated antibodies (Kirkegaard and Perry Laboratories, Gaithersburg, MD), were visualized with enhanced chemiluminescence reagent (Perkin Elmer, Boston, MA).

### Analysis of apoptosis

Annexin V-fluorescein isothiocyanate (FITC)/PI double staining was used to measure the percentile of apoptosis. Briefly, the cells were treated with curcumin and/or As^3+^ for 24 h. The cells were digested with 0.25% trypsin/EDTA followed by re-suspension in binding buffer and addition of Annexin V-FITC/PI. The apoptotic cells were measured using flow cytometry.

### Subcellular localization of the EGFP-mCherry-LC3 fusion protein

Images of live cells were taken and processed on an Olympus BX53 fluorescence microscope (Pittsburgh, PA). When autophagosomes accumulate (neutral pH), both red and green LC3 punctae are observed. The merging of these two colors generates a yellow color, indicating that autophagosomes are not fused with lysosomes to form autolysosomes and that the autophagy is not completed. When the fusion occurs and autolysosomes are generated (acidic pH), green fluorescence decreases and red is maintained. The net merged color will be more toward red and less toward yellow.

### Plasmid transfection

Transfection was performed using Lipofectamine™ 3000 (Invitrogen) according to the manufacturer’s protocol. Briefly, cells were seeded in 6-well culture plates and transfected with 4 μg plasmid at ~ 70% confluence. Expression of transfected protein was measured by immunoblotting 48 h post-transfection.

### As^3+^-induced cell transformation

BEAS-2B cells were treated with 0.5 μM As^3+^, 6.25 μM curcumin and/or 10 nM Baf1a for 36 weeks. The medium was replaced with fresh medium every 3 days. After 36 weeks, 1 × 10^4^ cells were suspended in 2 mL culture medium containing 0.35% agar, seeded into 6-well plates with a 0.5% agar base layer, and maintained in an incubator for 4 weeks. The cells were stained with 1 mg/mL iodonitrotetrazolium violet, and colonies greater than 0.1 mm in diameter were scored by microscope examination. As^3+^-transformed cells from anchorage-independent colonies were picked and continued to grow in DMEM. Passage-matched cells without As^3+^ treatment were used as controls.

### Xenograft tumor model

Six-week-old female athymic nude mice were purchased from Charles River Laboratories (Wilmington, MA). The mice were housed in sterilized filter-topped cages and maintained in a pathogen-free animal facility at the Chandler Medical Center, University of Kentucky. All of the animals were handled according to the Institutional Animal Care and Use Committee (IACUC) guidelines. BEAS-2B (1 × 10^6^ cells) in 100 μl of a mixture of 1 × DMEM and Matrigel (BD Biosciences) were subcutaneously (s.c.) injected into the left flank of each mouse. Tumors were measured using an external caliper and the volume was calculated using the formula: (length × width^2^)/2. After 21 days, the mice were euthanized, and the tumors were harvested for western blotting and immunofluorescence.

### Hematoxylin and eosin (HE) staining

Mouse tissue sections were deparaffinized, rehydrated, and rinsed, and hematoxylin was added. After rinsing and decolorization, the sections were counterstained in eosin and mounted with cytoseal. The sections were visualized using an Olympus BX53 fluorescence microscope.

### Immunohistochemical staining

Tumor tissues were fixed with 4% paraformaldehyde at room temperature for 24 h, embedded in paraffin, and sectioned (3–4 μm thickness). The slides were deparaffinized and rehydrated. Sections were incubated in indicated primary antibody (1: 400) for 24 h at 4 °C, washed in PBS, and incubated with secondary anti-rabbit-HRP antibody. Immuno-staining was visualized after 3–3′diamino-benzidine (DAB) staining according to the VECTASTAIN ABC kit protocol (Vector Laboratories, Burlingame, CA). The figure was obtained using bright field microscopy (Leica, Wetzlar, Germany). Five sections were selected from each animal and analyzed using the Image J software (National Institutes of Health).

### Statistical analysis

Statistical analyses were performed using GraphPad Prism 6.5 software (GraphPad Software, San Diego, CA, USA). Data from more than two groups were subjected to one-way analysis of variance (ANOVA) followed by Bonferroni’s *post-hoc* pairwise comparisons, whereas data between two groups were subjected to Student’s t-tests. Data are presented as the means ± SD. Significance was defined as *P* < 0.05.

## Results

### Curcumin acts as a dual-regulator of Nrf2 in normal and transformed cells

A previous study demonstrated that Nrf2 plays dual roles in carcinogenesis. In the early stages of cell transformation, activation of inducible Nrf2 decreased carcinogenesis. However, after cell transformation, inhibition of constitutively activated Nrf2 can be anti-oncogenic [24]. To develop the potential prevention agents, the antioxidant natural compounds were selected and screened using human lung bronchial epithelial BEAS-2B cells and As^3+^-induced transformed BEAS-2B cells (AsT).

**Fig. 1:**
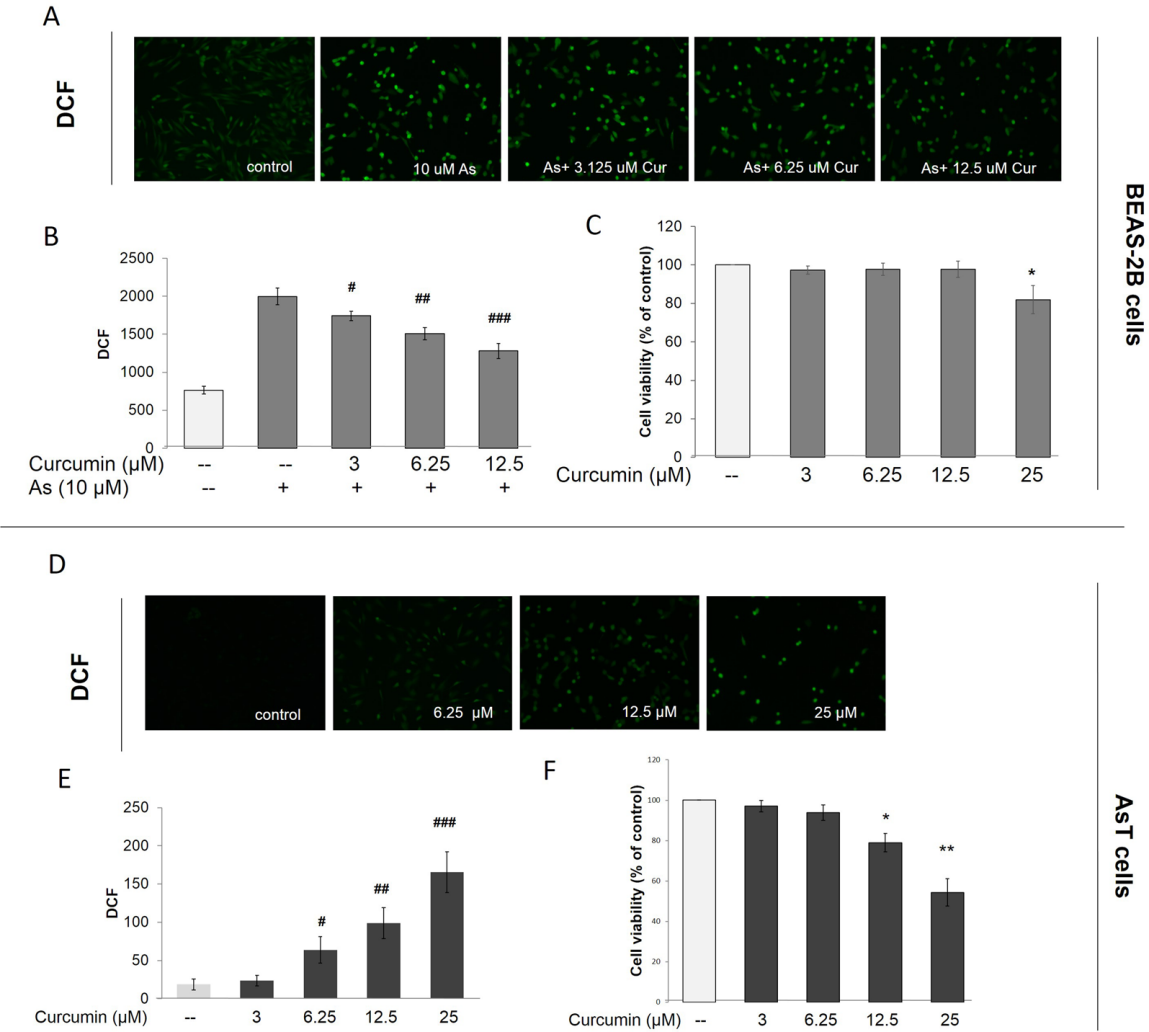
Curcumin decreased ROS in As^3+^ treated BEAS-2B cells, but increased ROS in As^3+^-induced transformed BEAS-2B cells. BEAS-2B cells were exposed to As^3+^ (10 μM) with or without curcumin (0, 3.125, 6.25, 12.5 μM) for 24 h and then were labeled with DCFDA (10 μM). **A** Images were taken with fluorescence microscopy and **B** fluorescent intensity determined by flow cytometry. **C** Cell viabilities were detected by staining with MTT assay. As^3+^-induced transformed BEAS-2B cells (AsT) were treated with curcumin (0, 3.125, 6.25, 12.5, 25 μM) for 24 h and then were labeled with DCFDA (10 μM). **D** Images were taken with fluorescence microscopy and **E** fluorescent intensity determined by flow cytometry. **F** Cell viabilities were detected by staining with MTT assay. The data are expressed as the mean ± SD of three independent experiments (n = 9). *p < 0.05, **p < *0.01* vs control. ^*#*^*p* < *0.05, *^*##*^*p* < *0.01* vs As^3+^ treatment only group

The chemopreventive potential of curcumin was demonstrated by its ability to induce antioxidant enzymes, including GST, NQO1, and HO-1 [2, 13, 20]. Growing evidence indicates that curcumin induces the Nrf2-mediated cellular defense system by modifying Keap1. Clinical studies indicate that curcumin has limited therapeutic potential because of its poor bioavailability. However, the long-term potential of curcumin in the prevention of heavy metal-induced carcinogenesis is unknown. In this study, we stained BEAS-2B cells with CM-H2DCFDA (5-(and-6)-chloromethyl-2′,7′-dichlorodihydrofluorescein diacetate, acetyl ester) to measure intracellular ROS levels. We also analyzed fluorescence intensity using fluorescence microscopy and flow cytometry (Fig. 1). ROS were measured after BEAS-2B cells were challenged by As^3+^ with or without curcumin treatment. In Fig. 1A, B, As^3+^ significantly induced ROS generation in BEAS-2B cells (B2B cells) up to a concentration of 10 μM for 24 h treatment, whereas curcumin itself showed decreased ROS (Fig. 1B). Compared to untreated cells, treatment with 3.125–25 μM curcumin caused a decrease in ROS in response to As^3+^. Further studies showed that the basal levels of ROS were lower in the AsT cells than in the non-transformed cells (Fig. 1B, E). Interestingly, the levels of ROS in AsT cells were upregulated by curcumin treatment in a dose-dependent manner (Fig. 1D, E). The results of cell viability assays showed that the safe concentration (12.5 μM) of curcumin could induce cell death (Fig. 1F). Taken together, the results suggest that although curcumin could prevent As^3+^-induced ROS, it induced ROS in transformed cells. Moreover, the specific inhibition effects of transformed cells but not normal cells indicated that curcumin has the potential to prevent As^3+^-induced carcinogenesis through regulating ROS.

### Curcumin induces Nrf2 by inhibiting the Keap1-Nrf2 interaction

**Fig. 2:**
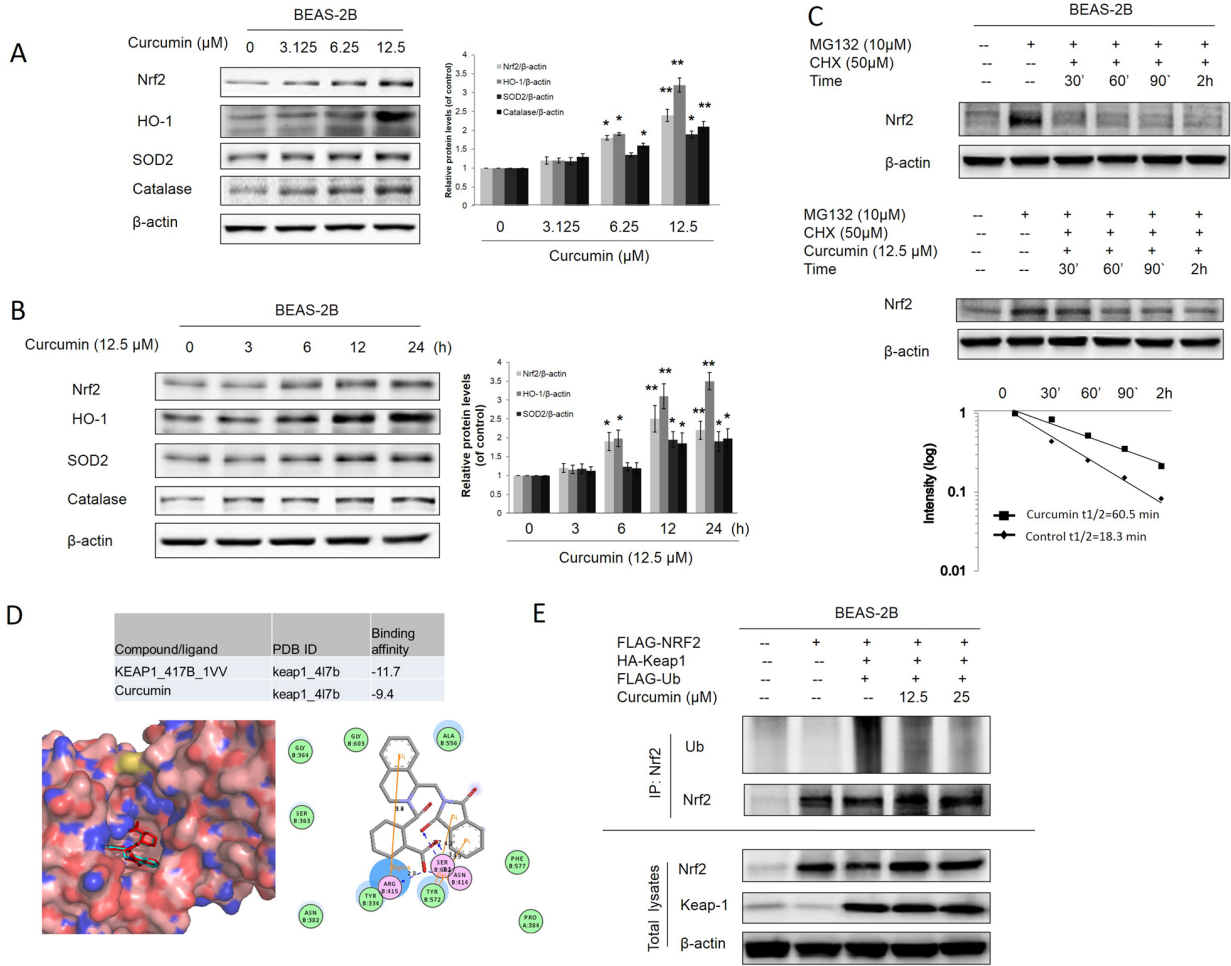
Curcumin induced Nrf2 expression by blocking Keap1-Nrf2 interaction. **A** Normal BEAS-2B cells were treated with (3.125, 6.25, 12.5 μM) curcumin for 24 h. **B** BEAS-2B cells were treated with 12.5 μM curcumin for 3, 6, 12 and 24 h. Cells were harvested and whole protein lysates were extracted. Nrf2, HO-1, SOD2 and catalase expression were examined by immunoblotting. **C** BEAS-2B cells were left untreated or were pretreated with 10 μM MG132 for 2 h after which cells were washed and treated with 50 μM CHX either in presence or absence of 12.5 μM curcumin. At different incubation times, cells were lysed and protein levels were evaluated by western blot using Nrf2 specific antibody. Expression of β-actin was evaluated as a loading control. **D** Curcumin directly bind to the ligand-binding site of Keap1 (PDB code 4L7B). Three-dimensional diagram displays the interaction of curcumin (the yellow stick) and the crystal ligand (the red sticks) to the ligand-binding site of Keap1. Two-dimensional diagram shows the interactions of curcumin to the amino acid residues (e.g., Tyr334, Asn387, Arg415, Ser508. Ser602) in the binding pocket of Keap1. Colors of the residues indicate the forms of interactions with distances as follows: van der Waals forces, green; polarity, magenta. Blue arrows represent H-bonding with the side chain of the amino acid residue. **E** BEAS-2B cells were co-transfected with expression vectors encoding FLAG-tagged Nrf2 and HA-tagged Keap1. 24 h after transfection cells were then treated with 6.25 or 12.5 μM curcumin along with MG132 (10 μM) for 2 h before cell lysates were collected for ubiquitination assay. Anti-Nrf2 immunoprecipitates were analyzed by immunoblot with anti-ubiquitin antibodies for detection of ubiquitin-conjugated Nrf2. The data are expressed as the mean ± SD of three independent experiments (n = 9). *p < 0.05, **p < *0.01* vs control

ROS production has been reported in various cellular systems exposed to As^3+^ at various concentrations, including in human lung bronchial epithelial BEAS-2B cells [23]. A previous study demonstrated that Nrf2 is antioncogenic in the early stages of As^3+^-induced cell transformation via the upregulation of antioxidants to reduce As^3+^-induced ROS [24]. Many studies have reported that curcumin exhibits antioxidant effects by activating the Nrf2 pathway [26, 27]. In this study, BEAS-2B cells were treated with curcumin, and western blotting data showed that curcumin in concentration-dependent and time-dependent Nrf2, HO-1, and antioxidant enzymes SOD2 and catalase expression (Fig. 2A, B). To investigate the mechanism by which curcumin activates Nrf2, we tested the ability of curcumin to modulate Nrf2 ubiquitylation as Nrf2 stability is regulated through ubiquitin mediated proteasomal degradation. Analyses of protein levels by western blot confirmed the short half-life of Nrf2, whose expression levels were barely detectable after 90 min of cycloheximide (CHX) treatment in untreated BEAS-2B cells. In contrast, co-treatment with curcumin clearly prolonged Nrf2 stability for up to 3 h of treatment, indicating that curcumin affects Nrf2 expression levels by inhibiting protein degradation (Fig. 2C). As shown in Fig. 2D, BEAS-2B cells were transferred expression vectors for FLAG-tagged Nrf2 (F-Nrf2), HA-tagged Keap1 (H-Keap1) and HA-Ubiquitin (H-Ub). The results indicated that curcumin concentration-dependently blocked ubiquitylation of Nrf2 (Fig. 2E). The results of docking analysis further confirmed that curcumin prevented the keap1 and Nrf2 interaction by binding the ligand-binding site of Keap1 (PDB code 4L7B). Taken together, these results indicate that curcumin activates the Nrf2-mediated defensive response by blocking the interaction of keap1 and ubiquitin, thus enhancing Nrf2 stability.

### Curcumin inhibited As^3+^-induced oxidative stress by activating Nrf2

**Fig. 3:**
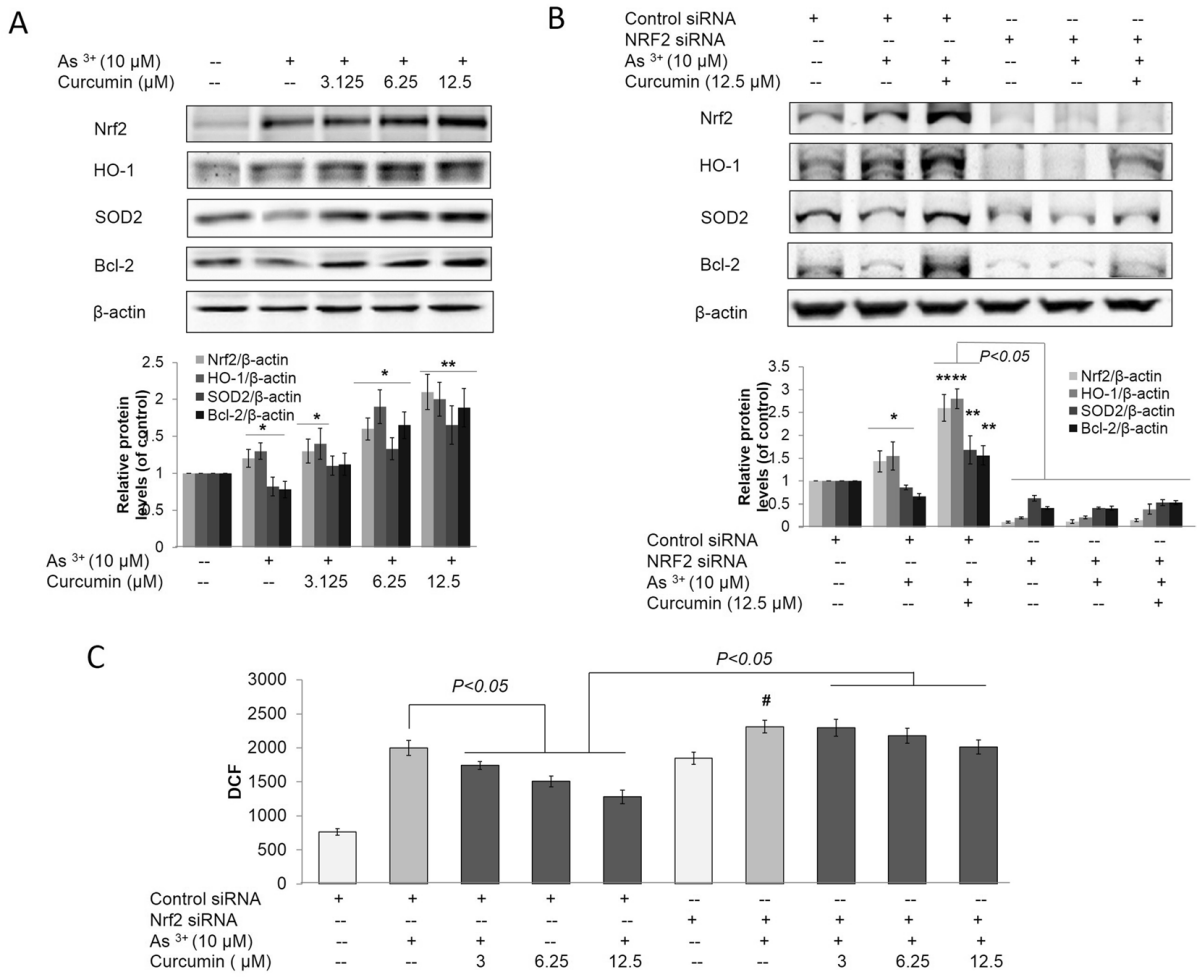
Curcumin alleviated As^3+^-induced oxidative stress by activating Nrf2. BEAS-2B cells were treated with 3.125, 6.25, 12.5 and 25 μM curcumin and/or 10 μM As^3+^ for 12 h. ROS levels were detected by DCF staining and detected by microscope and flow cytometry. **A** Normal BEAS-2B cells were treated with 3.125, 6.25 and 12.5 μM curcumin and/or 10 μM As^3+^ for 24 h. The treatment cells were harvested and whole protein lysates were extracted. Nrf2, HO-1, SOD-2 and Bcl-2 expression was examined by immunoblotting. **B** Normal BEAS-2B cells were transfected with either control-siRNA or Nrf2-siRNA for 48 h. BEAS-2B cells were then treated with 3.125, 6.25 and 12.5 μM curcumin and/or 10 μM As^3+^ for 24 h. The treatment cells were harvested and whole protein lysates were extracted. Nrf2, HO-1, SOD-2 and Bcl-2 expression was examined by immunoblotting. **C** DCF-based fluorescence was measured using flow cytometry. Cell lysates were subjected to immunoblot analysis. The data are expressed as the mean ± SD of three independent experiments (n = 9). *p < *0.05*, **p < *0.01* versus normal BEAS-2B control group

Since the protective effect of curcumin on oxidative stress, owing to its ability to induce Nrf2-mediated antioxidant, has been demonstrated [28], we showed that curcumin enhanced Nrf2 protein, HO-1, and SOD2 expression in response to As^3+^ (Fig. 3A). We propose that the protection against As^3+^-induced ROS by curcumin in BEAS-2B cells is due to the activation of Nrf2. To study the mechanism, the effects of curcumin on As^3+^-induced ROS in BEAS-2B cells with knockdown (KD) of Nrf2 were examined. Interestingly, curcumin-mediated suppression of ROS and promotion of Nrf2, HO-1, and SOD2 expression were not observed in cells in which Nrf2 expression was blocked by Nrf2-siRNA (Fig. 3B, C). This finding indicates that the protection of As^3+^-induced oxidative stress conferred by curcumin is Nrf2-dependent.

### Curcumin enhanced autophagic flux in As^3+^ treated BEAS-2B cells

**Fig. 4:**
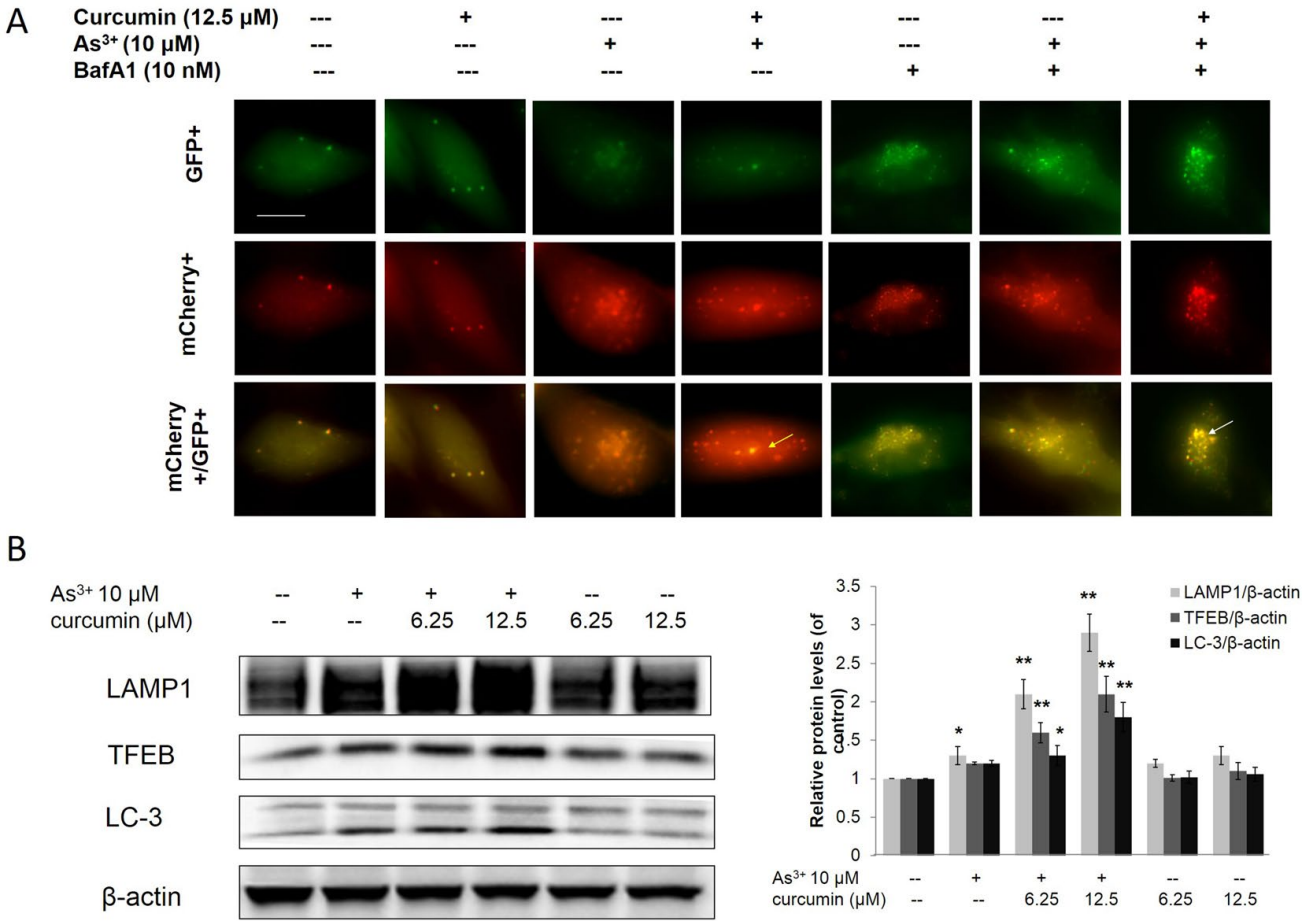
Curcumin increased As^3+^ -induced autophagy in normal BEAS-2B cells. **A** The mCherry-GFP-LC3 reporter is red and green when it associates with phagophores and autophagosomes. After fusion with lysosomes to form autolysosomes, the GFP signal is quenched by the acidic condition. The normal BEAS-2B (**A**) transfected with the mCherry-EGFP-LC3 construct and treated with As^3+^ and/or curcumin for 24 h. White arrows indicate autophagosome, yellow arrows indicate autolysosome. **B** Normal BEAS-2B cells were treated with 6.25, 12.5 μM curcumin and/or 10 μM As^3+^ for 24 h. The treatment cells were harvested and whole protein lysates were extracted. LAMP1, TFEB and LC-3II expression was examined by immunoblotting. The data are expressed as the mean ± SD of three independent experiments (n = 9). *p < *0.05*, **p < *0.01* versus normal BEAS-2B control group

A previous study reported that activation of autophagy could inhibit transformation and prevent cancer [29]. In this study, we transfected the tandem fluorescence protein mCherry-GFP-LC3 construct into BEAS-2B cells to track cell autophagy in real time. As shown in Fig. 4A, when the BEAS-2B cells were treated with As^3+^, both red and green LC-3 puncae were observed. The merged color was more toward red and less toward yellow, indicating that autophagosomes were generated and were fused with lysosomes to generate autolysosomes, and that BEAS-2B cells are autophagy capable. In the curcumin treatment alone group, both red and green LC-3 puncae were observed with low intensities. The merged color was more toward yellow and less toward red. These results show that only a limited number of autophagosomes were generated and that they were not fused with lysosomes to generate autolysosomes, demonstrating that curcumin alone is insufficient to induce autophagy. However, curcumin increased the As^3+^-induced autophagy in BEAS-2B cells, as shown by the increased intensities of red LC-3 puncae and by the change in the merged color toward red. Bafilomycin A1 (Baf A1), a H + ATPase (V-ATPase) inhibitor, prevents maturation of autophagic vacuoles by inhibiting fusion between autophagosomes and lysosomes. As shown in Fig. 4A, Baf A1 treatment led to a significant accumulation of both red and green LC-3 puncae and changed the merged color toward yellow. Interestingly, curcumin enhanced As^3+^-induced autophagy was attenuated by treatment with Baf A1. Transcription factor EB (TFEB) and lysosomal-associated membrane protein 1 (LAMP-1) are master factors for lysosomal formation. The results from western blotting demonstrated that curcumin enhanced As^3+^-induced LC-3II, TFEB, and LAMP1 expression, but no change was observed with curcumin treatment alone (Fig. 4B). These results show that curcumin increased As^3+^-induced autophagy by enhancing autophagic flux in As^3+^ treated BEAS-2B cells.

### Curcumin induced autophagy by activating Nrf2-mediated p62/LC-3 complex formation and enhanced autophagic flux in As^3+^ treated BEAS-2B cells

**Fig. 5:**
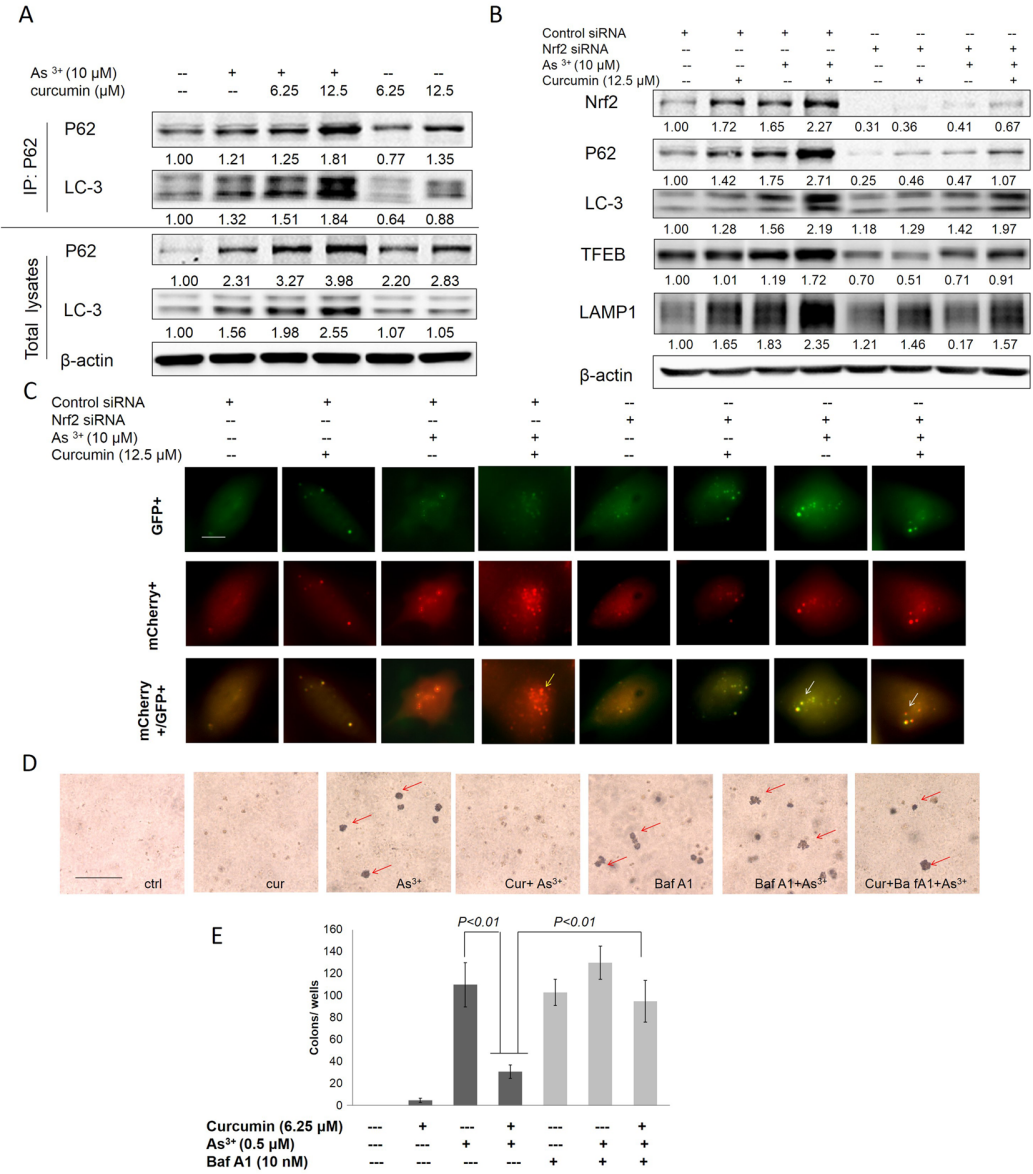
Curcumin inhibits chronic As^3+^-induced malignant transformation via enhancing autophagy in normal BEAS-2B cells. **A** BEAS-2B cells were treated with 6.25, 12.5 μM curcumin and/or 10 μM As^3+^ for 24 h before cell lysates were collected. Anti-p62 immunoprecipitates were analyzed by immunoblot with anti-p62 and anti-LC-3 antibodies for detection of P62-conjugated LC-3, whole protein lysates were collected and p62, LC-3 and β-actin expression were examined by immunoblotting. **B** BEAS-2B cells were transfected with control siRNA and Nrf2 siRNA. 24 h after transfection cells were then treated with 6.25, 12.5 μM curcumin and/or 10 μM As^3+^ for 24 h before cell lysates were collected. Nrf2, p62, LAMP1, TFEB and LC-3 expression were examined by immunoblotting. **C** BEAS-2B cells were co-transfected with the mCherry-EGFP-LC3 construct and Nrf2 siRNA. 24 h after transfection cells were then treated with As^3+^ and/or curcumin for 24 h. Yellow arrows indicate autolysosome (red). **D** BEAS-2B cells were maintained in a medium containing As^3+^ (0 or 0.5 μM) with or without 6.25 μM curcumin for 6 months. Cells were cultured in 0.35% soft agar for 5 weeks. **E** Colony numbers in the entire dish were counted. The data are expressed as the mean ± SD of three independent experiments (n = 9)

p62 interacts directly with microtubule-associated protein 1 light chain 3 (LC3), a component of the autophagosomal membrane, which is cleaved (LC3-I) and lipidated (LC3-II) and can be used as a marker for autophagy [30]. In Fig. 5, the Co-IP results revealed that normal BEAS-2B cells treated with curcumin could enhance p62 and LC-3 expression. The increased in p62 led to recruitment of LC-3 to form the P62/LC-3 complex in a dose-dependent manner (Fig. 5A). To investigate the link between Nrf2 and autophagy, Nrf2 was knocked down in normal BEAS-2B cells by Nrf2 siRNA. The results revealed that curcumin induced Nrf2, P62, LC-3, and autophagic flux factors TFEB and LAMP-1, and the formation of the P62/LC-3 complex, which were significantly reversed by knockdown of Nrf2 in normal BEAS-2B cells (Fig. 5B). Furthermore, normal BEAS-2B cells were co-transfected with the mCherry-EGFP-LC3 construct and Nrf2 siRNA. The results indicated that knockdown of Nrf2 significantly blocked autophagy through reducing the numbers of lysosomes and autolysosomes following As^3+^ and/or curcumin treatment (Fig. 5C). Taken together, these results suggest that curcumin enhanced autophagy in As^3+^ treated normal BEAS-2B cells via Nrf2-mediated p62/LC-3 complex formation and expression of autophagic flux factors TFEB and LAMP1.

### Curcumin inhibits malignant transformation induced by chronic As^3+^ exposure by enhancing autophagy in BEAS-2B cells

Previous reports have demonstrated that chronic As^3+^ exposure to BEAS-2B cells results in malignant transformation, as assessed by increased cell proliferation, and anchorage independent growth in soft agar [24]. In the current study, malignant transformation was assessed by anchorage-independent growth in soft agar [31]. Continuous exposure of BEAS-2B cells to 0.5 μM As^3+^ for 6 months induced a marked increase in the size and number of colonies compared to the untreated control (Fig. 5D, E). However, co-treatment of curcumin (6.25 μM) with As^3+^ significantly decreased anchorage independent growth in soft agar. According to the autophagy effect of curcumin in As^3+^ treatment, we propose that the inhibition of colony forming effect of curcumin may be related to its autophagic property. Indeed, blockade of the autophagic flux by Bafa1 showed that the inhibition effect of curcumin in colony formation was significantly reversed by co-treatment with curcumin, Bafa1 with As^3+^. To our surprise, treatment with Bafa1 alone could also induce colony formation. These results further confirm that curcumin inhibited malignant transformation induced by chronic As^3+^ exposure through enhancing autophagy in BEAS-2B cells.

### Curcumin increased ROS and cell death in AsT by downregulating Nrf2

**Fig. 6:**
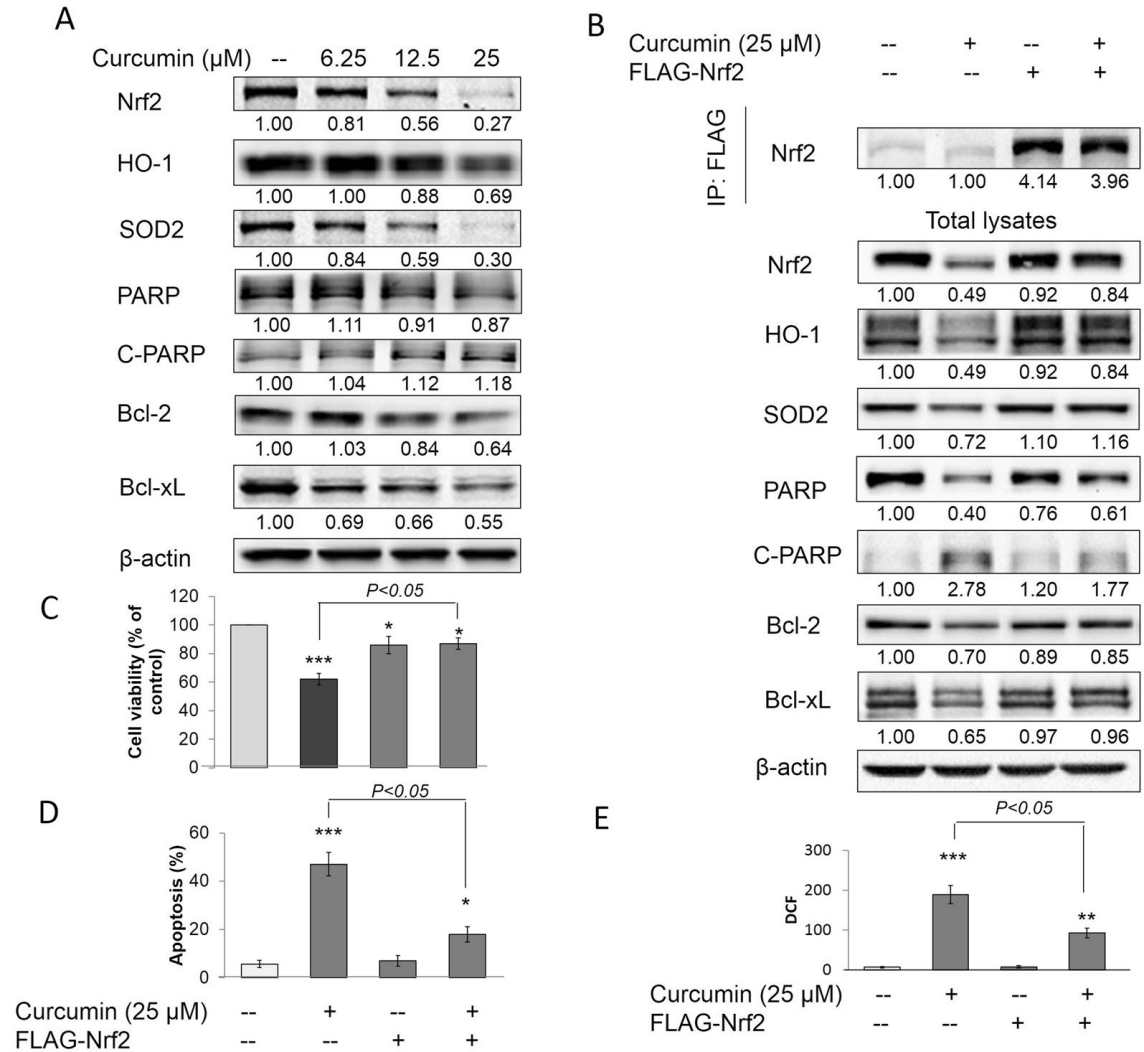
curcumin induced ROS and cell death in AsT by downregulating Nrf2. **A** AsT cells were treated with 6.25, 12.5, 25 μM curcumin for 24 h before cell lysates were collected. Nrf2, HO-1, SOD2, PARP, cleaved PARP, Bcl-2, Bcl-xl and β-actin expression were examined by immunoblotting. **B** AsT cells were transfected with FLAG-Nrf2. 24 h after transfection cells were then treated with 25 μM curcumin for 24 h before cell lysates were collected. Anti-FLAG immunoprecipitates were analyzed by immunoblot with anti-Nrf2 antibody for detection of the transfection expression levels of Nrf2, whole protein lysates were collected and Nrf2, HO-1, SOD2, PARP, cleaved PARP, Bcl-2, Bcl-xl and β-actin expression were examined by immunoblotting. AsT cells were transfected with FLAG-Nrf2. 24 h after transfection cells were then treated with 25 μM curcumin for 24 h before cells were collected. **C** Cell viabilities were detected by staining with MTT assay, **D** apoptosis rates were stained PI/Annex V and analyzed with flow cytometry. **E** ROS levels were detected with DCF and analyzed with flow cytometry. The data are expressed as the mean ± SD of three independent experiments (n = 9). *p < *0.05*, **p < *0.01* versus AsT control group

Previous studies have demonstrated that AsT cells have cell death resistance properties. A low level of ROS is caused by high expression of antioxidant enzymes, and high expression of Bcl-2 and Bcl-xL are involved in the cell death resistance mechanisms of AsT cells [24]. In this study, AsT cells were treated with curcumin for 24 h, and the results showed that curcumin significantly reduced Nrf2, HO-1, SOD2, PARP, Bcl-2, and Bcl-xl expression, but increased cleaved PARP levels in a concentration-dependent manner (Fig. 6A). Further studies showed that curcumin decreased cell viability (Fig. 6C), increased cell apoptosis (Fig. 6D), and upregulated the levels of ROS of AsT cells. These data indicated that curcumin induced ROS generation and cell death via reducing antioxidant enzymes and inducing apoptosis in AsT cells. Interestingly, curcumin reduced the expression of Nrf2, HO-1, SOD2, PARP, Bcl-2, and Bcl-xl, and decreased cell viability, increased cell apoptosis, and upregulated ROS, all of which were significantly abolished by overexpression of Nrf2 in AsT cells (Fig. 6B–E). These results demonstrated that curcumin induced ROS and cell apoptosis in AsT by downregulating Nrf2.

### Curcumin decreased Nrf2 by activating GSK3β but independent of Nrf2/Keap1 in AsT cells

**Fig. 7:**
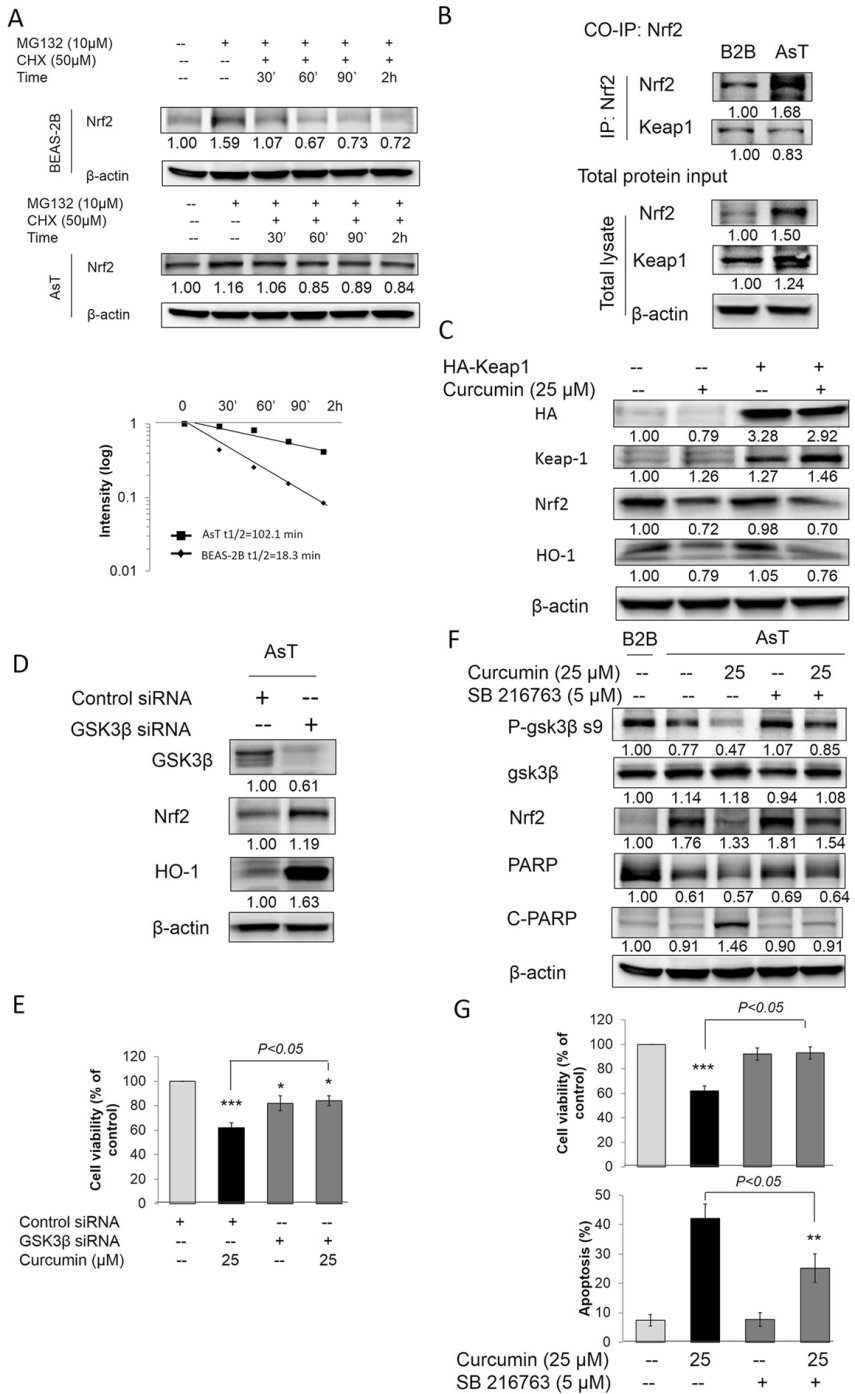
Curcumin decreased Nrf2 by activating GSK3β but independent of Nrf2/Keap1 in AsT cells. **A** BEAS-2B or AsT cells were untreated or were pretreated with 10 μM MG132 for 2 h after which cells were washed and treated with 50 μM CHX. At different incubation times, cells were lysed and protein levels were evaluated by western blot using Nrf2 specific antibody. Expression of β-actin was evaluated as a loading control. **B** BEAS-2B or AsT cells were treated with 10 μM MG132 for 2 h before cell lysates were collected. Anti-Nrf2 immunoprecipitates were analyzed by immunoblot with anti-Nrf2 and anti-Keap1 antibodies for detection of Nrf2-conjugated Keap1, whole protein lysates were collected and Nrf2, Keap1 and β-actin expression were examined by immunoblotting. **C** AsT cells were transfected with HA-Keap1. 24 h after transfection cells were then treated with 25 μM curcumin for 24 h before cell lysates were collected. HA, Keap1, Nrf2 and HO-1 expression were examined by immunoblotting. **D** AsT cells were transfected with control and GSK3β siRNA. After 24 h transfection, cell lysates were collected. GSK3β, Nrf2 and HO-1 expression were examined by immunoblotting. **E** AsT cells were transfected with control and GSK3β siRNA. 24 h after transfection cells were then treated with 25 μM curcumin for 24 h. Cell viabilities were detected by staining with MTT assay. AsT cells were co-treated with 25 μM curcumin and 5 μM SB216763 for 24 h. Cell lysates were collected and p-GSK3β s9, GSK3β, Nrf2, PARP and cleaved PARP expression were examined by immunoblotting (**F**). Cell viabilities and cell apoptosis were detected by staining with MTT assay and PI/Annex V assay, individually (**G**). The data are expressed as the mean ± SD of three independent experiments (n = 9). *p < 0.05, **p < *0.01* vs control

It has been reported that a constitutively high level of Nrf2 in AsT cells upregulates the antioxidant proteins catalase and superoxide dismutase, the consequences of which are decreased ROS generation and increased apoptotic resistance, cell survival, proliferation, and tumorigenesis [24]. Normally, Nrf2 is principally controlled through protein ubiquitylation, which targets it for proteasomal degradation. The N-terminal Nrf2-ECH homology (Neh)2 domains of Nrf2 contain an ETGE motif to which Kelch-like ECH-associated protein-1 (Keap1) binds with high affinity. Keap1 is a dimeric protein that jointly serves as a ubiquitin ligase adaptor and substrate receptor and induces Nrf2 proteasomal degradation [32]. In our unpublished results, we found that Nrf2 cannot bind with Keep1 due to the mutation of the ETGE motif of the Neh2 domain of Nrf2 (data not shown). In this study, we found that the degradation of Nrf2 in AsT cells was slower than that in normal BEAS-2B cells (Fig. 7A). The results of Co-IP assay further confirmed that Nrf2 cannot bind with Keep1 in AsT cells, but can in normal BEAS-2B cells (Fig. 7B). Overexpression of Keap-1 in AsT cells by transferred HA-Keap vectors cannot reduce Nrf2 and HO-1 levels (Fig. 7C). These results indicate that curcumin decreased Nrf2 in As^3+^-transformed cells independent of Nrf2/Keap1.

As constitutive activation of Nrf2 in tumors is associated with increased death resistance and a higher rate of cell proliferation, it is desirable to identify Keap1-independent mechanisms [33, 34]. Now, evidence has shown that GSK-3-catalyzed phosphorylation of the Neh6 domain in Nrf2 creates a phosphodegron to which the substrate receptor β-transducin repeat-containing protein (β-TrCP) is recruited and induces Nrf2 proteasomal degradation. Treatment with the GSK-3 inhibitor SB216763 has been shown to result in increased Nrf2 and HO-1 levels in the liver and hippocampus [15, 35]. To extend these investigations, we knocked down or pharmacologically inhibited GSK with GSK-3β siRNA or SB216763 in AsT cells. The results showed that knockdown of GSK-3β significantly increased Nrf2 and HO-1 expression in AsT cells (Fig. 7D). Furthermore, knockdown of GSK-3β reversed the curcumin-induced reduction in cell viability of AsT cells (Fig. 7E). Pharmacologic inhibition of GSK-3β activity by treatment with SB216763 showed that the activation of GSK-3β significantly activated by for treating curcumin; however, SB216763 can inhibit the activation effects of curcumin on GSK-3β by upregulating p-GSK-3β s9. Moreover, SB216763 can also reverse the reduction in Nrf2 and PARP induced by curcumin, and increased the levels of cleaved-PARP in AsT cells (Fig. 7F). The decreased cell viability and increased cell apoptosis of AsT cells induced by curcumin was also reversed by SB216763 treatment (Fig. 7G). These data demonstrate that inhibition of GSK3β could increase Nrf2 and decrease apoptosis in curcumin-treated AsT cells.

### Curcumin induced Nrf2 degradation by mediating the interaction of Nrf2 with the GSK-3β/β-TrCP axis

**Fig. 8:**
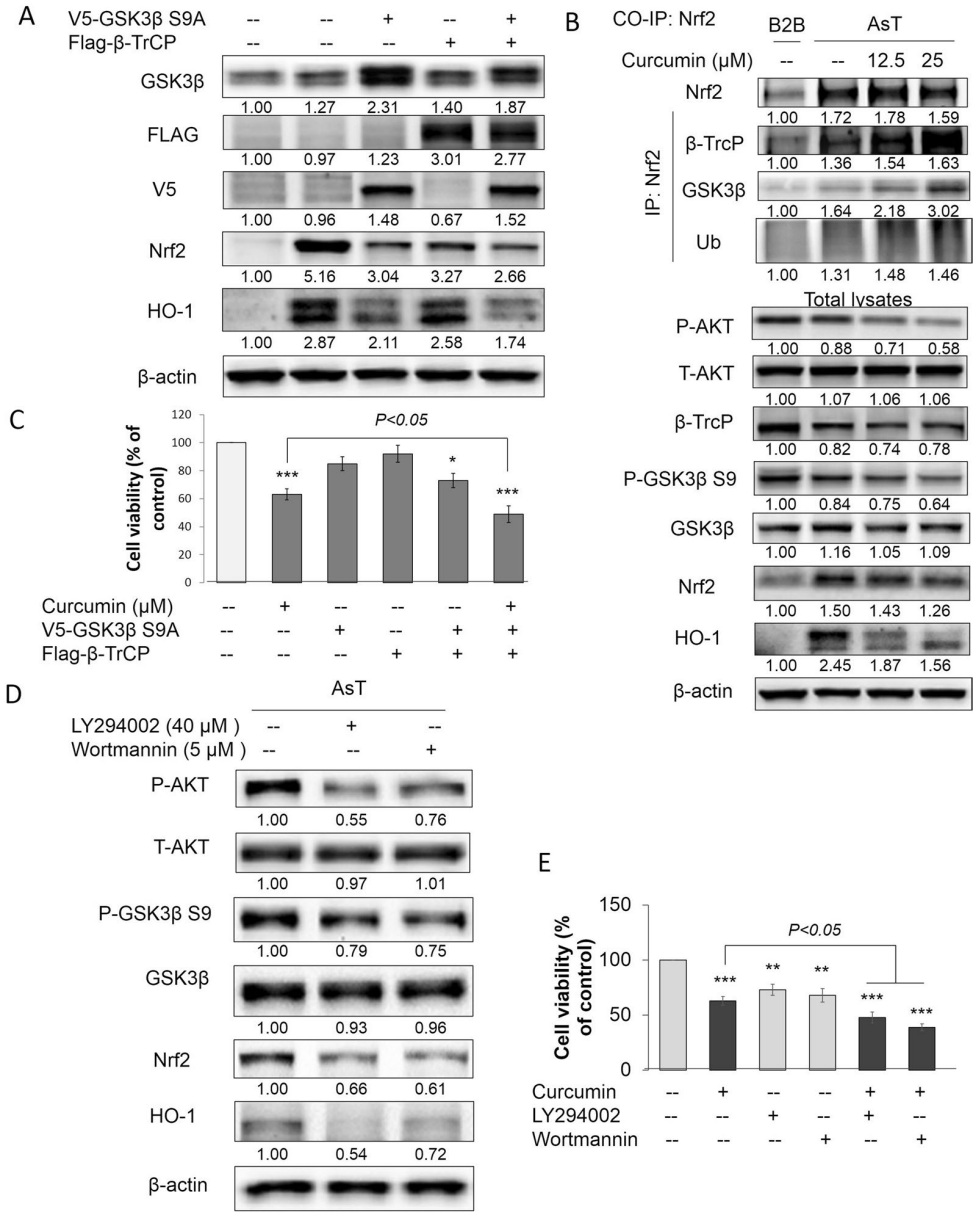
Curcumin induced Nrf2 degradation by mediating the interaction of Nrf2 with the GSK-3β/β-TrCP axis. **A** AsT cells were transfected with V5-GSK3β S9A and/or Flag-β-TrCP for 24 h. V5, FLAG, GSK3β, β-TrCP, Nrf2 and HO-1 expression were examined by immunoblotting. **B** AsT cells were treated with 12.5 and 25 μM curcumin for 24 h before cell lysates were collected. Anti-Nrf2 immunoprecipitates were analyzed by immunoblot with anti-Nrf2 and anti-GSK3β, anti-β-TrCP and anti-ubiquitin antibodies for detection of Nrf2-conjugated GSK3β, β-TrCP and ubiquitin, whole protein lysates were collected and p-Akt Ser-473, Akt, p-GSK3β S9, GSK3β, β-TrCP, Nrf2 and HO-1 and β-actin expression were examined by immunoblotting. **C** AsT cells were transfected with V5-GSK3β S9A and/or Flag-β-TrCP for 24 h. Cell viabilities were detected by staining with MTT assay. AsT cells were treated with 40 μM LY294002 or 5 μM wortmannin for 24 h. **D** Cell lysates were collected and p-Akt Ser-473, Akt, p-GSK3β S9, GSK3β, Nrf2 and HO-1 and β-actin expression were examined by immunoblotting. **E** Cell viabilities were detected by staining with MTT assay. The data are expressed as the mean ± SD of three independent experiments (n = 9). *p < 0.05, **p < *0.01* vs control

To evaluate the role of GSK3β in curcumin treated AsT cells, we established AsT cells stably expressing constitutive-active (S9A) GSK3β mutants. The S9A mutant is resistant to inhibitory regulation by restraining phosphorylation at Ser9, and has been previously shown to effectively stimulate GSK3β activity [36]. Overexpression of GSK3β S9A or β-TrCP proteins were verified by the expression of V5 or Flag tag by immunoblotting (Fig. 8A). We demonstrated that activation of GSK3β and overexpression of β-TrCP reduced Nrf2 and HO-1 expression (Fig. 8A). Meanwhile, overexpression of GSK3β S9A induces cell death and enhanced curcumin-induced AsT cell death. The interaction between Nrf2 and β-TrCP is necessary for the ubiquitylation of Nrf2 bypassing Keap-1 [15, 35]. The Co-IP assay results, involving pulldown of Nrf2 in curcumin-treated AsT cells, showed that curcumin induced β-TrCP, GSK3β, and ubiquitin binding to Nrf2 in a concentration-dependent manner. The total lysates demonstrated that curcumin could inhibit the phosphorylation of AKT and GSK3β S9A and decrease the protein levels of Nrf2 and HO-1 (Fig. 8B). Previous studies have shown that the reduction in Nrf2 protein levels is caused by phosphorylation of GSK3β at Ser-9 and the inactivation of phosphorylation Akt at Ser-473 [33]. We next inhibited the PI3K/Akt signaling pathway using LY294002 or wortmannin. Figure 8C shows that inhibition of Akt activity activated GSK3β by increasing the phosphorylation of GSK3β S9A and reducing the levels of Nrf2 and HO-1 proteins. The cell viability results also showed that inhibition of the PI3K/Akt signaling pathway in AsT cells could induce cell death and enhance curcumin-induced cell death (Fig. 8D, E). These results indicate that curcumin induced Nrf2 degradation by mediating the interaction between Nrf2 and the GSK-3β/β-TrCP axis, and that curcumin activated GSK3β by inhibiting the AKT signaling pathway in AsT cells.

### Curcumin inhibited angiogenesis of AsT cells through modulating Nrf2-mediated HIF1α/VEGF signaling pathway

**Fig. 9:**
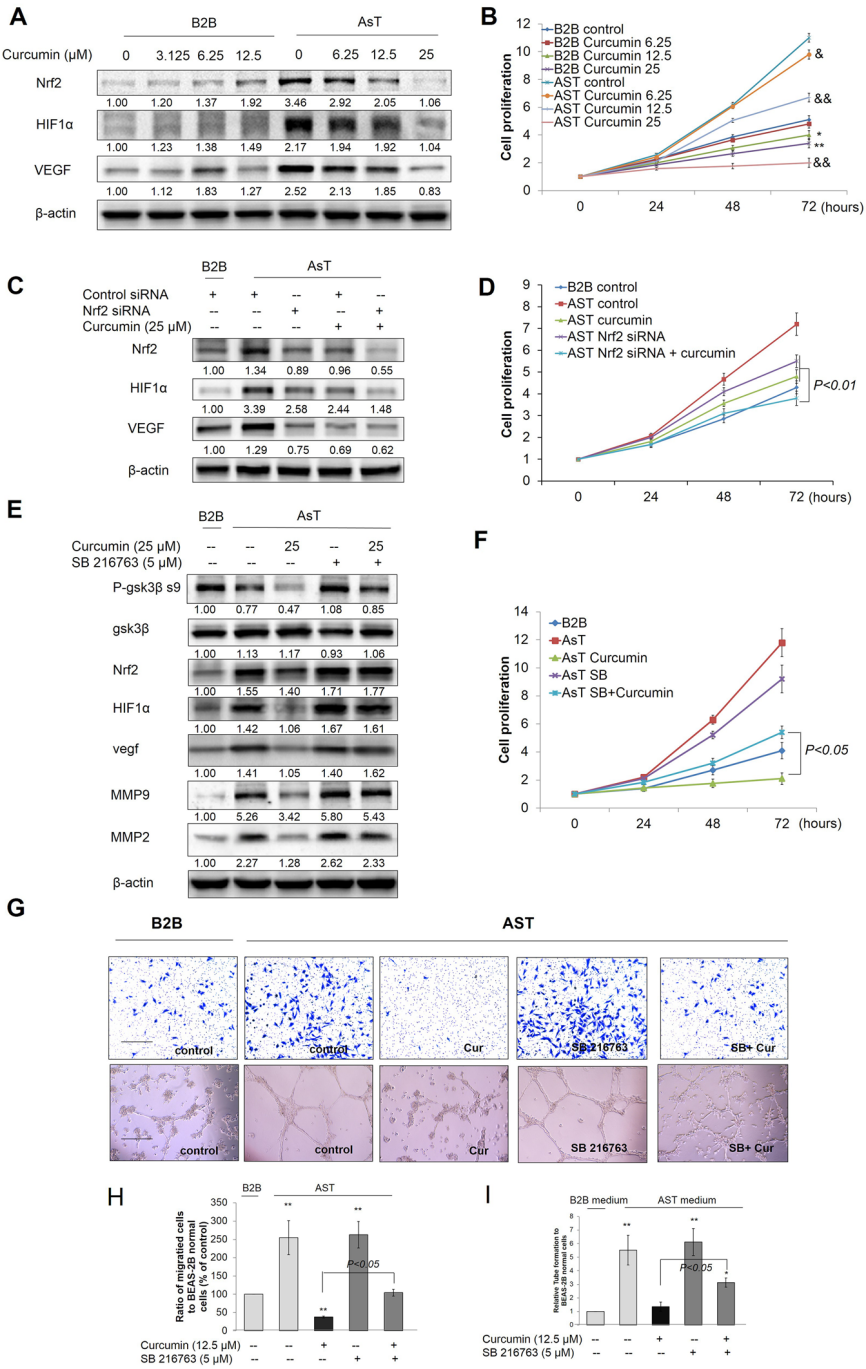
Curcumin suppressed angiogenesis through modulating Nrf2-mediated HIF1α/ VEGF signaling pathway in AsT cells. **A** AsT cells and their parent non-transformed BEAS-2B cells were exposed to increasing concentrations (3.125, 6.25, 12.5 μM) of curcumin for 24 h, the levels of Nrf2, HIF1α and VEGF were detected by western blotting. **B** AsT cells and their parent non-transformed BEAS-2B cells were exposed to increasing concentrations (6.25, 12.5, 25 μM) of curcumin for 24, 48 and 72 h. Cell proliferations were detected by MTT assay. **C** AsT cells were transfected with control siRNA or Nrf2 siRNA for 24 h prior to 25 μM curcumin treatment for 24 h. the levels of Nrf2, HIF1α and VEGF were detected by western blotting and **D** Cell proliferations of 24, 48 and 72 h curcumin (25 μM) treatment were detected by MTT assay. AsT cells were treated with 5 μM SB216763 and/or 25 μM curcumin for 24 h, **E** the levels of p-GSK3β s9, GSK3β, Nrf2, HIF1α, VEGF and MMP2/9 were detected by western blotting. **F** Cell viabilities were detected by staining with MTT assay. **G** Cell invasion were detected by Transwell Migration Assay. HUVECs were incubated in 5 μM SB216763 and/or 25 μM curcumin treated AsT cells culture medium and tube formation were detected by Matrigel assay. The quantitative analysis of migration (**H**) and tube formation (**I**). The data are expressed as the mean ± SD of three independent experiments (n = 9). *p < 0.05 *versus* normal BEAS-2B control group; ^&^*p* < 0.05, ^&&^*p* < 0.01 *versus* AsT cells control group

Previous studies and our own have shown that chronic exposure to heavy metals, such as As^3+^ and cadmium, induces malignant transformation and stimulates the growth of tumors through a HIF1α-dependent stimulation of angiogenesis [37, 38]. Knockdown of Nrf2 suppresses angiogenesis by decreasing HO-1 and vascular endothelial growth factor (VEGF) expression and inhibiting HIF1α [39, 40]. To determine the effects of curcumin on angiogenesis signaling molecules in AsT cells, we first investigated whether curcumin treatment changes the expression of HIF1α and VEGF, important pathways regulating tumor angiogenesis. Normal BEAS-2B and AsT cells were exposed to curcumin for 24 h, and the effects on Nrf2, HIF1,α and VEGF signaling were analyzed by immunoblotting. Interestingly, the expression of Nrf2, HIF1α, and VEGF was induced by curcumin treatment in normal BEAS-2B cells in a concentration-dependent manner, whereas their expression was inhibited by curcumin treatment in AsT cells (Fig. 9A). Results from the cell viability assay showed that curcumin inhibited cell proliferation in AsT cells, but had no effects on normal BEAS-2B cells (Fig. 9B). To further determine whether Nrf2 is necessary for curcumin-mediated suppression of angiogenesis in AsT cells, AsT cells were transfected with control siRNA or Nrf2 siRNA for 24 h before 25 μM curcumin treatment for 24 h. Western blotting results showed that knockdown of Nrf2 enhanced curcumin-decreased Nrf2, HIF1α, and VEGF in AsT cells (Fig. 9C). Furthermore, curcumin inhibited cell proliferation of AsT was also enhanced by knockdown of Nrf2 (Fig. 9D). These results indicate that curcumin suppressed angiogenesis of AsT cells through inhibition of the Nrf2-mediated HIF1α/VEGF signaling pathway.

We next sought to determine the role of GSK3β in modulating the anti-angiogenic effects of curcumin in AsT cells. To this end, AsT cells were treated with 5 μM SB216763 and/or 25 μM curcumin for 24 h. Western blotting results showed that inhibition of GSK3β reversed curcumin-decreased p-GSK3β s9, Nrf2, HIF1α, VEGF, and MMP2/9 expression in AsT cells (Fig. 9E). Furthermore, curcumin-inhibited cell proliferation, migration, and tube formation were also reversed by inhibition of GSK3β (Fig. 9F–I). Taken together, curcumin-suppressed angiogenesis through inhibition of the Nrf2-mediated HIF1α/VEGF signaling pathway in AsT cells.

### Inhibition of GSK3β activity reversed the inhibition of colony formation and carcinogenesis development in curcumin in AsT cells

**Fig. 10:**
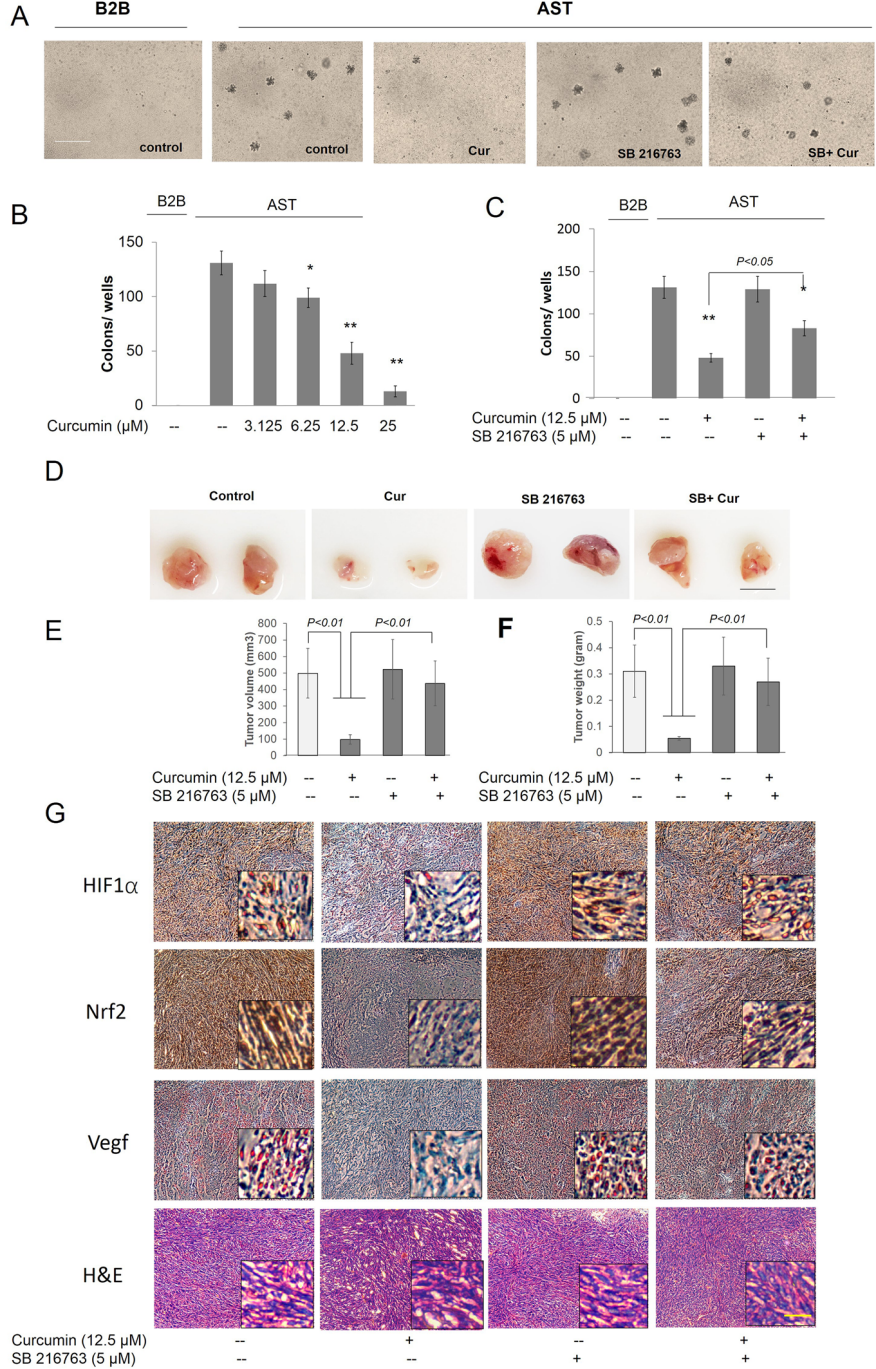
Inhibition of GSK3β activity reversed the inhibition of colony formation and carcinogenesis development in curcumin in AsT cells. **A** BEAS-2B cells were maintained in a medium containing As^3+^ (0 or 0.5 μM) for 9 months, after As^3+^-induced BEAS-2B cells transformed, the transformation cells were treated with or without curcumin (**B**) and/or 5 μM SB216763 (**C**) for 1 week. Cells were cultured in 0.35% soft agar for 5 weeks. Colony numbers in the entire dish were counted. **D** AsT cells treated as indicated from different treatments were injected into the flanks of 6-week old athymic nude mice (1 × 10^6^ cells per mouse) and checked daily for tumor appearance; tumor volume (**E**) and weight (**F**) was measured after 21 days injected. Tumor volume was determined by Vernier caliper, following the formula A × B2 × 0.52, where A is the longest diameter of tumor and B is the shortest diameter. The data are expressed as the mean ± SD of three independent experiments (n = 9). *p < 0.05, **p < 0.01 *versus* AsT cells control group. **G** Angiogenic (HIF1α and VEGF) markers and Nrf2 were decreased in tumors treated with both As^3+^ and curcumin, while inhibition of GSK3β could reverse the inhibition effects of curcumin as evident from immunohistochemistry. Frozen tumor Sections (5 μM thick) were subjected to immunoperoxidase staining (dark brown) to detect HIF1α, VEGF, and Nrf2 expressions. The data are expressed as the mean ± SD of three independent experiments (n = 9). *p < 0.05, **p < *0.01* vs control

Over a 9-month period, BEAS-2B cells were exposed to As^3+^, and the colony numbers gradually increased compared to the vehicle control (Fig. 10A). Moreover, treatment with curcumin significantly decreased the number of As^3+^-induced colonies (Fig. 10A, B). However, inhibition of GSK3β by co-treatment of curcumin and SB216763 could reverse the curcumin-induced inhibition of colony formation (Fig. 10A, C). The xenograft assay also demonstrated that the injection of BEAS-2B cells could not generate tumor in animals, while tumors could be generated by injection of 9-month As^3+^-exposed BEAS-2B cells (Fig. 10D). However, the tumor growth significantly decreased in the 9-month As^3+^-exposed BEAS-2B cells following treatment with curcumin (Fig. 10D). To us expect, inhibition of GSK3β by SB216763 could reverse the curcumin-induced inhibition of tumor growth (Fig. 10D–F). The high expression levels of Nrf2, HIF1α, and VEGF in As^3+^-exposed-induced tumor samples were further confirmed by immunohistochemistry (Fig. 10G). Moreover, Nrf2, HIF1α, and VEGF were significantly reduced in curcumin-treated As^3+^-exposed induced tumor samples, while inhibition of GSK3β could reverse the inhibitory effects of curcumin treatment (Fig. 10G). The malignancy of tumor tissue was confirmed by hematoxylin and eosin (H&E) staining (Fig. 10G).

## Discussion

As^3+^ has been shown to be associated with multiple cancer types, including lung, bladder, kidney, liver, and skin cancers, in numerous clinical studies [41–44]. As^3+^ was also shown to be significantly associated with lung cancer incidence rates in the U.S. [45]. Previous studies have shown that chronic exposure of human BEAS-2B cells to As^3+^ generates ROS, and that ROS is responsible for As^3+^-induced transformation of these cells [23, 24]. In our study, we demonstrated that curcumin could significantly decrease As^3+^-generated ROS by activating Nrf2 and its target antioxidant genes. Moreover, curcumin induced autophagy in As^3+^-treated BEAS-2B cells by inducing autophagy via formation of the p62/LC-3 complex, and increasing autophagic flux by promoting TFEB and LAMP1 expression. Knockdown of Nrf2 abolished curcumin-induced autophagy and ROS downregulation. Further studies showed that inhibiting fusion between autophagosomes and lysosomes with bafilomycin a1 (BafA1) could block curcumin-prevented As^3+^-induced cell transformation. These results demonstrate that curcumin prevented As^3+^-induced cell transformation by inducing autophagy via activating the Nrf2 signaling pathway in BEAS-2B cells. Previous studies have demonstrated that constitutively high levels of Nrf2 in As^3+^-transformed AsT cells caused low levels of ROS and induced apoptotic resistance, cell survival, angiogenesis, and tumorigenesis [23, 24]. In our study, we demonstrated that curcumin inhibited constitutive expression of Nrf2 and enhanced ROS and apoptosis in AsT cells to prevent apoptotic resistance, cell survival, angiogenesis, and tumorigenesis.

ROS production has been reported in various cellular systems, including human BEAS-2B cells, exposed to As^3+^ at various concentrations [23]. The Keap1-Nrf2 system is under investigation for the development of protein–protein interaction inhibitors that will stabilize Nrf2 for therapeutic effect in conditions of inflammation and cancer [6]. To develop small-molecule inhibitors of the Keap1-Nrf2 interaction, molecular docking was used to virtually screen natural antioxidant chemical databases and identify molecules that interact with the ligand-binding site of Keap1 (PDB code 4L7B). The cell-based assays and molecular docking results revealed that curcumin has significantly inhibitory activity against Keap1-4L7B. Moreover, the Co-IP results indicate that curcumin is a potent Keap1 Kelch domain-dependent Nrf2 activator that stabilizes Nrf2 by hindering its ubiquitination. The increased activation of Nrf2 and its target antioxidant genes following curcumin treatment could significantly decrease As^3+^-generated ROS.

Autophagy is a degradation pathway that is essential for survival during starvation, hypoxia, immune responses, and chemotherapy exposure [46]. An increasing number of studies have reported that activation of autophagy can inhibit transformation and prevent cancer [29, 47]. The process of autophagy is involved in the following mechanisms: (i) the recruitment of the LC3-p62 complex assembled during formation of the autophagosome; (ii) induction of lysosomal biogenesis and lysosome function; and (iii) promotion of the fusion of autophagosomes with lysosomes to induce basal autophagy [48]. On the initiation of autophagy, LC3 is processed from LC3-I (a clear molecular weight of 16 kDa) to LC3-II (14 kDa). The levels of LC3-II are proportional to the number of accumulated autophagosomes [49]. In the present study, curcumin enhanced As^3+^-induced LC-3II expression, which was not observed following curcumin treatment alone, indicating that curcumin could enhance autophagy of BEAS-2B cells (Fig. 4B). We then transfected the tandem fluorescence protein mCherry-GFP-LC3 construct into BEAS-2B cells to track cell autophagy in real time. The results were consistent with those of western blotting, in that curcumin enhanced autophagic flux in As^3+^ BEAS-2B cells by inducing lysosome formation and promoting autophagosomal–lysosomal fusion, which was not observed with curcumin treatment alone (Fig. 4A). The basic helix-loop-helix leucine zipper transcription factor TFEB has emerged as a master gene that regulates the number and function of lysosomes and autophagy [50]. TFEB promotes autophagosomal–lysosomal fusion, a process commonly referred to as maturation or degradation; this transcription factor can resolve the autophagic buildup and maintain the degradation pathway. LAMP1 is an important factor that regulates the number and function of lysosomes and autophagy [51]. Bafilomycin A1 (Baf A1) is an inhibitor of autophagic flux by blocking H + ATPase (V-ATPase) and prevents maturation of autophagic vacuoles by inhibiting fusion between autophagosomes and lysosomes. Further study demonstrated that curcumin enhanced As^3+^-induced autophagic flux, and that LC-3II, TFEB, and LAMP1 expression were attenuated by treatment with Baf A1, further confirming that curcumin induced autophagy in As3 + treated BEAS-2B cells by enhancing autophagic flux. Initiation of autophagy via the LC3-p62 complex resulted in autophagosome formation. SQSTM1/p62 is a polyubiquitin binding protein that is regulated by Nrf2 and degraded by autophagy, thereby displaying an inverse relationship with the level of autophagic activity. In this study, BEAS-2B cells treated with curcumin could enhance p62 and LC-3 expression. To investigate the link between Nrf2 and autophagy for treating curcumin, Nrf2 was knocked down in normal BEAS-2B cells by Nrf2 siRNA. The results revealed that curcumin induced expression of Nrf2, P62, and LC-3, and autophagic flux factors TFEB and LAMP-1, as well as formation of the P62/LC-3 complex, all of which were significantly reversed by knockdown of Nrf2 in normal BEAS-2B cells. These results further demonstrated that curcumin enhanced autophagy in As^3+^ treated normal BEAS-2B cells via two-step activation of Nrf2. The first step is the induction of the p62/LC-3 complex formation to initiate autophagy by inducing autophagosome formation, and the second step is promoting autophagic flux via stimulating TFEB and LAMP1 expression. We hypothesize that the promotion of autophagy could prevent the transformation of normal cells. Figure 5D, E demonstrates that continuous exposure of normal BEAS-2B cells to 0.5 μM As^3+^ for a 6-month period induced a marked increase in the size and number of colonies compared to untreated control cells. However, the growth of transformed cells on agar was dramatically inhibited by co-treatment with curcumin. The inhibition of autophagy reversed the effect of curcumin, further confirming that curcumin prevents As^3+^-induced cell transformation by promoting cell autophagy, including the initiation of autophagy by formation of the p62/LC-3 complex and enhanced autophagic flux by stimulating TFEB and LAMP1 expression.

Nrf2 has antioncogenic properties and can upregulate antioxidant enzymes to reduce ROS, a process that is considered as the first stage of cell transformation. Curcumin prevents cell transformation in this stage by upregulating Nrf2 and inducing antioxidant enzymes to reduce ROS. However, following transformation (e.g., the post-malignant stage), ROS play an antioncogenic role. A low level of ROS increases survival and proliferation of transformed cells and tumorigenesis. In this stage, constitutive overexpression of Nrf2 has oncogenic properties. The constitutive overexpression of Nrf2 further upregulates antioxidant enzymes and antiapoptotic proteins in the transformed cells, promoting cell survival, proliferation, and carcinogenesis of transformed cells [24]. Previous studies have led us to question how curcumin affects the second stage of As^3+^-induced cell transformation. Indeed, curcumin could mediate a concentration-dependent decrease in cell viability and increase in ROS levels in AsT cells. However, surprisingly, the regulation of Nrf2 protein levels in AsT cells was found to be Keap1-independent, as evidenced by the finding that overexpression of Keap-1 resulted in constitutively high levels of Nrf2 in AsT cells. Constitutive overexpression of Nrf2 in AsT cells demonstrated that curcumin increased ROS levels and induced cell apoptosis by decreasing Nrf2. To establish how curcumin decreases Nrf2 in AsT cells, we focused on the Keap1-independent pathway of Nrf2 regulation. Further study showed that curcumin decreased the Nrf2 level in AsT cells by activating GSK-3β and inhibiting PI3K/AKT activation. The link between curcumin-decreased Nrf2 and GSK-3β was confirmed by Co-IP assay results, which showed that curcumin promoted the interaction of Nrf2 with the GSK-3β/β-TrCP axis and ubiquitin. Moreover, the inhibition of GSK-3β reversed Nrf2 in curcumin-treated AsT cells, indicating that the decrease in Nrf2 by curcumin was due to activation of the GSK-3β/β-TrCP ubiquitination pathway.

Inhibition of angiogenesis is an attractive approach to treat cancer [52]. Previous studies have indicated that As^3+^ promotes angiogenesis via an Nrf2-mediated HO-1-dependent mechanism and HIF-1α [53]. HIF-1α is involved in tumorigenesis promoting effects in many tumors [54]. In our study, treatment with As^3+^ elevated the expression of HIF-1α, VEGF, and MMP-2/9, and promoted angiogenesis, all of which were markedly suppressed by curcumin treatment. Knockdown of Nrf2 enhanced the curcumin-induced decrease in Nrf2, HIF1α, and VEGF in AsT cells (Fig. 9C). Moreover, the proliferation (Fig. 9D) of AsT cells indicated that curcumin suppressed angiogenesis of AsT through inhibiting the Nrf2-mediated HIF1α/VEGF signaling pathway. We next sought to determine the role of GSK3β in the anti-angiogenic effects of curcumin in AsT cells. We showed that inhibition of GSK3β reversed curcumin decreased p-GSK3β s9, Nrf2, HIF1α, VEGF, and MMP2/9 in AsT cells (Fig. 9E). Interestingly, curcumin-inhibited cell proliferation, migration, and tube formation were also reversed by inhibition of GSK3β (Fig. 9F–I), indicating that curcumin suppressed angiogenesis through inhibition of the GSK3β/Nrf2-mediated HIF1α/VEGF signaling pathway in AsT cells. The xenograft and colony assays further confirmed that the anti-carcinogenesis effects of curcumin were due to regulation of the GSK-3β/β-TrCP ubiquitination/Nrf2 pathway.

Curcumin, a major yellow pigment and spice in turmeric and curry, is a powerful anti-cancer agent. The anti-tumor activities of curcumin include inhibition of tumor proliferation, angiogenesis, invasion, and metastasis, induction of tumor apoptosis, increase in chemotherapeutic sensitivity, and regulation of cell cycle and cancer stem cells, indicating that curcumin has strong therapeutic potential in modulating the progression of various cancers. Numerous studies have provided evidence that curcumin protects against As^3+^-induced neurotoxicity, genotoxicity, and DNA damage in vivo and in vitro [55–57]. To date, more than 100 clinical trials have been completed with curcumin, all of which have demonstrated its safety, tolerability, and effectiveness against various chronic diseases, including various cancers, diabetes, obesity, and cardiovascular, pulmonary, neurological, and autoimmune diseases in humans [58]. Curcumin may represent a useful supplement to improve chronic inflammation and prevent carcinogenic changes in patients [59]. Although some issues with the bioavailability of curcumin have been reported in previous studies, numerous preclinical and clinical studies have reported success in combinatorial strategies, in which curcumin is coupled with other treatments. These studies have indicated that curcumin is a promising molecule for the prevention and treatment of cancer [60, 61].

## Conclusions

**Fig. 11:**
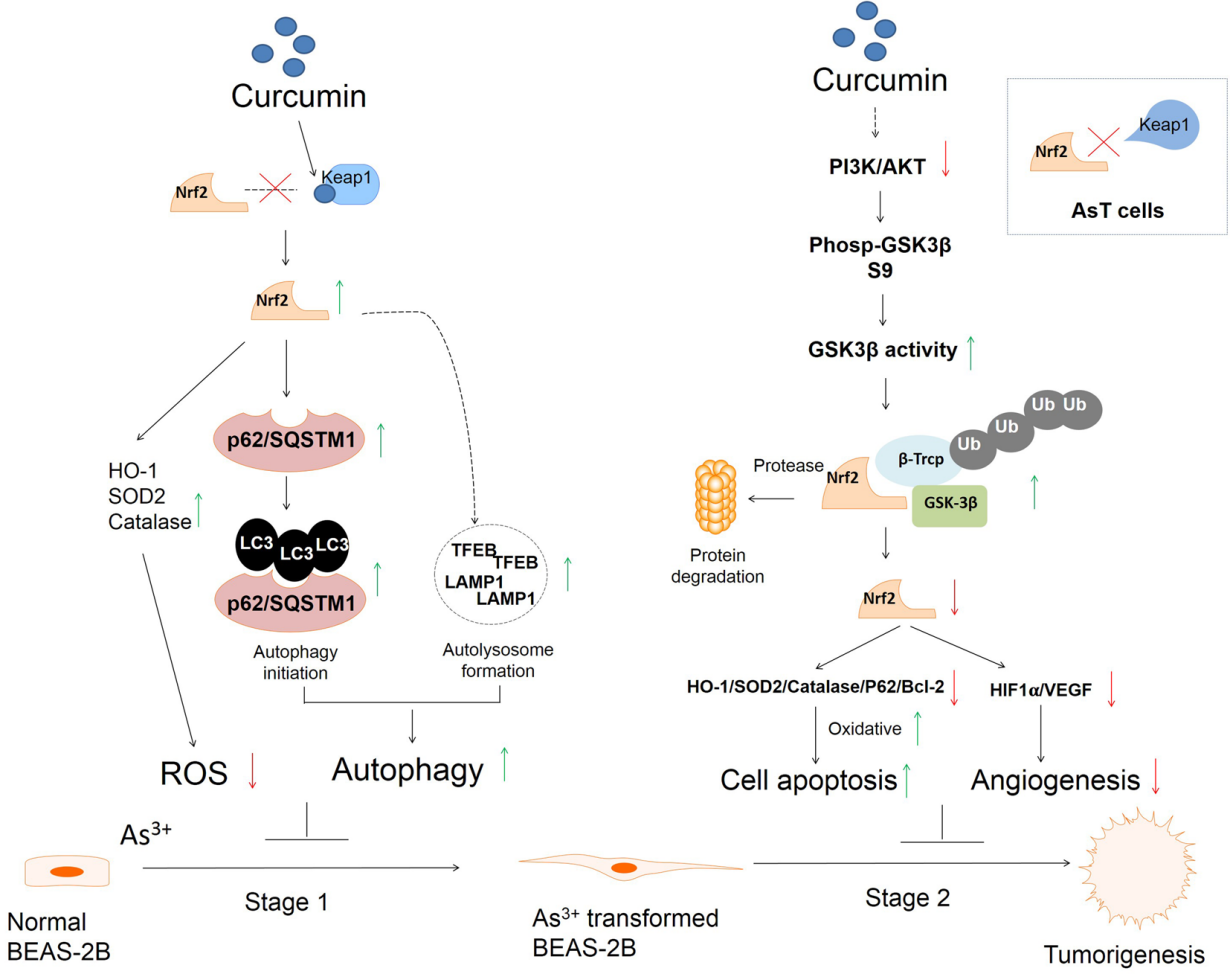
Proposed mechanism of curcumin inhibits As^3+^-induced cell transformation, carcinogenesis and tumorigenesis

In summary, in the first stage of As^3+^-induced carcinogenesis, curcumin activates Nrf2, decreases ROS, and induces autophagy in normal cells to prevent As^3+^-induced cell transformation. In the second stage, curcumin inhibits constitutive expression of Nrf2 and promotion of ROS, apoptosis, and inhibition of angiogenesis in AsT cells to prevent tumorigenesis (Fig. 11). Our results suggest that antioxidant natural compounds such as curcumin should be evaluated further as potential candidates for complementary therapy for As^3+^-induced carcinogenesis.

## Data Availability

The data used to support the current study are available from the corresponding author on reasonable request.
